# Chronic LCMV infection regulates the effector T cell response by inducing the generation of less immunogenic dendritic cells

**DOI:** 10.1038/s12276-023-00991-5

**Published:** 2023-05-01

**Authors:** Seungbo Yoo, Yun Hee Jeong, Hong-Hee Choi, Sehyun Chae, Daehee Hwang, Sung Jae Shin, Sang-Jun Ha

**Affiliations:** 1 Department of Biochemistry, College of Life Science and Biotechnology, Yonsei University, Seoul, 03722 Republic of Korea; 2 Brain Korea 21 (BK21) FOUR Program, Yonsei Education & Research Center for Biosystems, Yonsei University, Seoul, 03722 Republic of Korea; 3 Department of Microbiology, Institute for Immunology and Immunological Diseases, Brain Korea 21 PLUS Project for Medical Science, Yonsei University College of Medicine, Seoul, Republic of Korea; 4 Korea Brain Bank, Korea Brain Research Institute (KBRI), Daegu, 41062 Republic of Korea; 5 Department of Biological Sciences, Seoul National University, Seoul, 08826 Republic of Korea

**Keywords:** Infection, Lymphocyte differentiation

## Abstract

Chronic viral infection impairs systemic immunity in the host; however, the mechanism underlying the dysfunction of immune cells in chronic viral infection is incompletely understood. In this study, we studied the lineage differentiation of hematopoietic stem cells (HSCs) during chronic viral infection to elucidate the changes in dendritic cell (DC) differentiation and subsequent impact on T cell functionality using a chronic lymphocytic choriomeningitis virus (LCMV) infection model. We first investigated the lineage differentiation of HSCs in the bone marrow (BM) to elucidate the modulation of immune cell differentiation and found that the populations highly restrained in their differentiation were common myeloid progenitors (CMPs) and common dendritic cell progenitors (CDPs). Of interest, the main immune cells infected with LCMV Clone 13 (CL13) in the BM were CD11b/c^+^ myeloid DCs. We next characterized CD11b^+^ DCs that differentiated during chronic LCMV infection. These DCs displayed a less immunogenic phenotype than DCs in naive or acutely infected mice, showing low expression of CD80 but high expression of PD-L1, B7-H4, IDO, TGF-β, and IL-10. Consequently, these CD11b^+^ DCs induced less effective CD8^+^ T cells and more Foxp3^+^ regulatory T (Treg) cells. Furthermore, CD11b^+^ DCs generated during CL13 infection could not induce effective CD8^+^ T cells specific to the antigens of newly invading pathogens. Our findings demonstrate that DCs generated from the BM during chronic viral infection cannot activate fully functional effector CD8^+^ T cells specific to newly incoming antigens as well as persistent antigens themselves, suggesting a potential cause of the functional alterations in the T cell immune response during chronic viral infection.

## Virus infection: Chronic infection weakens immune cell response

Persistent virus infections impair the ability of the immune system’s dendritic cells to orchestrate a proper anti-pathogen defense, a finding that could lead to new treatments for chronic viral illnesses. Seungbo Yoo, Yun Hee Jeong, and Sang-Jun Ha of Yonsei University in Seoul, South Korea, and colleagues studied mice infected with lymphocytic choriomeningitis virus (LCMV), a virus carried by rodents that can cause neurological disease in humans. They showed that the virus localizes inside the bone-marrow of the animals, where it changes the way that dendritic cells form. These immunological sentinel cells normally present pieces of the viral intruder to virus-fighting T cells for them to then mount an attack. In LCMV-infected mice, the dendritic cells expressed fewer immune-stimulatory molecules and more inhibitory molecules, leading to weaker immune responses that allow for long-term viral persistence.

## Introduction

Chronic viral infections, including HIV, hepatitis B virus, and hepatitis C virus infections in humans and lymphocytic choriomeningitis virus (LCMV) infection in mice, suppress the host immune system, resulting in failure to protect against persistent infections by other types of pathogens^1,2^. The reasons for these ineffective antiviral immune responses under such conditions are not clear. Moreover, antiviral T cell responses are often unable to control viral replication, although effective T cell responses are crucial for the control of many viral infections. Under these conditions, poor control of chronic viral infection is associated with T cell exhaustion caused by viral persistence. Many studies on T cell exhaustion have been conducted recently; however, most of those studies have focused on the T cells themselves, specifically identifying exhaustion markers, defining subsets and studying epigenetic regulation in exhausted T cells, rather than clarifying the mechanism^3–5^. Although the mechanisms of T cell exhaustion associated with various environments and strategies for overcoming such deficits have also been studied^6–8^, the exhaustion and functional alterations of T cells in chronic viral infection remain to be further elucidated. For optimal activation and functioning of T cells, three adequate signals are needed: antigen (Ag) recognition, a costimulatory signal, and cytokine signaling. These signals can be acquired via interaction with dendritic cells (DCs). In this respect, to better understand functional changes in T cells during chronic viral infection, it is necessary to focus on the cells that interact with T cells, especially DCs and progenitor cells, which are considered possible reservoirs for pathogens.

DCs have been regarded as key mediators of innate and adaptive immunity. As mentioned above, DCs can prime T cells at the onset of an infection through a process in which multiple interactions occur with surface molecules. As antigen-presenting cells (APCs), DCs take up, process and present encountered antigens on major histocompatibility complex (MHC) molecules in peptide chain form. Then, the T cell receptor (TCR) on the T cell surface interacts with the Ag-MHC complex; thus, the first signal for T cell function is accomplished, which is essential for T cell action. DCs express not only MHC molecules but also various surface molecules that facilitate immune responses. Among those surface molecules, CD40, CD80 and CD86 have been designated as costimulatory molecules. These molecules act as costimulators by interacting with CD28 and CD40L on the T cell surface. This is how the second signal for T cell activation is obtained, and the absence of this signal results in T cell anergy. As immune cells, DCs can express various cytokines, including interleukins (ILs) and interferons (IFNs). Inflammatory cytokines, such as IL-6, IL-12 and tumor necrosis factor-alpha (TNF-α), secreted by DCs can augment T cell activation and function^9^. DCs can also contribute to immune suppression and act as central regulators of immune tolerance^10^. DCs can express inhibitory molecules, such as PD-L1, PD-L2, and PVR, and the interactions of these molecules with their ligands on T cells inhibit immune responses^11^. Furthermore, the expression of immunosuppressive cytokines, including IL-10 and TGF-β, by DCs can also repress immunity^12,13^. These immunoregulatory DCs have been called ‘tolerogenic DCs’^14^. To this end, phenotyping and characterization of these ‘Janus-faced’ dendritic cells should be performed to understand the mechanisms of T cell dysfunction during chronic viral infection.

Differentiation and development of immune cells occurs in the bone marrow (BM), after which lineage-differentiated immune cells migrate to lymphoid organs or other tissues. Hematopoietic stem cells (HSCs) can act as reservoirs for viruses^15^, and hematopoiesis is altered during viral infection^16,17^. However, it has been difficult to show established infection in these cells. Alternatively, recent studies have shown that multipotent progenitor (MPP) cells are susceptible to HIV infection, which leads to upregulation of PD-1 expression on monocytes that is correlated with high plasma concentrations of IL-10^18,19^.

Here, we determined whether chronic LCMV infects HSCs or progenitor cells and how these cells differentiate during chronic viral infection. As BM cells are thought to function as progenitors capable of regulating immunity, we hypothesized that the BM can be altered during chronic viral infection, thereby causing modified differentiation of immune cells, especially DCs, and functional alterations in T cells. In accordance with this concept, we confirmed that the BM serves as a long-term viral reservoir by determining the viral titer and staining with an antibody against the LCMV nucleocapsid protein (NP). Subsequently, we examined whether the immune system, including DCs in the BM, can be modulated during chronic infection with LCMV and whether this modulatory effect can contribute to the differentiation of T cells specific to either chronic viral antigens themselves or newly invading antigens, resulting in systemic immune alteration.

## Materials and methods

### Mice

Female C57BL/6 (B6) mice (5–6 weeks of age) were purchased from DBL (Chungcheongbuk-do, Korea) and Orient Bio (Gyeonggi-do, Korea). OT-I TCR-transgenic mice (C57BL/6-Tg(TcraTcrb)1100Mjb/J, RRID: IMSR_JAX:003831), which express a T cell receptor recognizing the ovalbumin (OVA) peptide residues 257-264 (OVA_257-264_) presented on H-2K^b^, and Ly5.1-expressing congenic mice (B6.SJL-*Ptprc*^*a*^ *Pepc*^*b*^/BoyJ, RRID: IMSR_JAX:002014) were purchased from the Jackson Laboratory. Purchased OT-I and Ly5.1-expressing mice were mated to generate Ly5.1^+^ OT-I mice. P14 TCR-transgenic B6 mice, which carry T cell receptors specific for the LCMV glycoprotein 33-41 peptide residues (GP_33-41_) in the context of H-2D^b^, and Thy1.1-expressing congenic B6 mice were generously provided by Rafi Ahmed of Emory University (Atlanta). These two strains were mated to generate Thy1.1^+^ P14 mice. All mice were maintained in a specific pathogen-free facility at the Yonsei Laboratory Animal Research Center (YLARC) of Yonsei University. All experimental procedures were performed in accordance with the YLARC Institutional Animal Care and Use Committee (IACUC) guidelines for the ethical use of animals.

### Virus

For LCMV infection, mice were intraperitoneally injected with 2 × 10^5^ plaque-forming units (PFU) of LCMV Armstrong (ARM) or intravascularly injected with 2 × 10^6^ PFU of LCMV Clone 13 (CL13). LCMV titers were determined by plaque assays, as described previously^20^. Vaccinia virus expressing OVA antigen (VV-OVA) was kindly provided by Hang-Rae Kim of Seoul National University College of Medicine. Mice were intraperitoneally injected with 2 × 10^6^ PFU of VV-OVA.

### Plaque formation assay

Vero cells were seeded in 6-well plates at a density of 2 × 10^5^ cells per well. The next day, the culture medium was removed, and 200 μl of serially diluted BM cell lysate harvested from LCMV CL13-infected mouse femurs was added to each well. After 1 hour of incubation at 37 °C, the cells in each well were overlaid with 4 ml of agarose mixture containing medium 199, FBS, penicillin‒streptomycin and L-glutamine. Four days later, 3 ml of 1% neutral red dye dissolved in the agarose mixture was added to each well. After 18 hours, the plaques that had formed in each well were counted before and after removing the agarose layers.

### Generation of BMDCs in vitro

To generate BMDCs in vitro, BM cells were isolated by flushing the femurs and tibias of naive or LCMV-infected mice with RPMI-1640 medium containing 2% FBS, and red blood cells were lysed with ACK lysis buffer (Gibco, Life Technologies). Purified BM cells were cultured at a density of 7.5 × 10^5^ cells/well in 6-well non-tissue culture plates in the presence of 20 ng/ml murine GM-CSF (Creagene, Korea). The cells were first cultured in 2 ml of medium, and 2 ml of fresh medium containing GM-CSF (20 ng/ml) was added on Day 4. On Day 7, 2 ml of preexisting medium was removed, and the same volume of fresh medium containing GM-CSF (20 ng/ml) was added. For DC stimulation, 2 ml of fresh medium containing 500 ng/ml LPS from *Escherichia coli* O111:B4 (Sigma Aldrich) was added on Day 9 after removing 2 ml of preexisting medium; then, the suspended cells were harvested on Day 10. DCs were cocultured with CellTrace™ Violet (Thermo Fisher)-labeled CD8^+^ P14 cells, OT-I T cells or CD4^+^ T cells.

### Ex vivo isolation of splenic DCs (SPDCs)

To manipulate CD11b^+^ myeloid DCs ex vivo, spleens were harvested from naive or LCMV-infected (10 days postinfection) mice. The spleens were enzymatically digested with 1 mg/ml type II collagenase D and 1 mg/ml DNase I. Red blood cells were lysed with ACK lysis buffer. CD19^+^, CD49b^+^, and CD90.2^+^ populations were primarily removed with a microbead cocktail (Miltenyi Biotec), and CD16/32 was blocked with a purified anti-CD16/32 antibody (Thermo Fisher). Then, CD11c^+^ cells were isolated with CD11c MicroBeads UltraPure (Miltenyi). For stimulation of DCs, 500 ng/ml LPS was added to the culture medium for 5 hours, and the stimulated DCs were cocultured with CellTrace™ Violet (Thermo Fisher)-labeled CD8^+^ P14 cells, OT-I T cells or CD4^+^ T cells.

### In vitro CD8^+^ T cell functional assay

Antigen-specific naive CD8^+^ T cells were isolated from the spleen of P14 TCR-transgenic mice and purified by negative selection using magnetic-activated cell sorting (Miltenyi Biotec). T cells were then labeled with 5 μM CellTrace™ Violet (Thermo Fisher) and cocultured for 3 days with either BMDCs or SPDCs at a ratio of 5:1 (500,000 T cells:100,000 DCs). For stimulation, 20 ng/ml GP_33-41_ peptide and GolgiPlug/GolgiStop™ (BD Bioscience) were added to the cultured cells for the last 5 hours. CD8^+^ OT-I T cells were isolated from the spleen of OT-I transgenic mice and purified by negative selection with MagniSort™ beads (Thermo Fisher). The T cells were then labeled with 5 μM CellTrace™ Violet (Thermo Fisher) and cocultured for 3 days with either BMDCs or SPDCs pulsed for 2 hours with the OVA protein (20 μg/ml) at a ratio of 5:1 (250,000 T cells:50,000 DCs). For stimulation, 0.5 μg/ml OVA_257-264_ SIINFEKL peptide and GolgiPlug/GolgiStop™ (BD Bioscience) were added to the cultured cells for the last 5 hours.

### In vitro Treg cell conversion assays

For the Foxp3^+^ regulatory T (Treg) cell conversion assay, the CD25^+^ population in harvested splenocytes from naive mice was primarily removed with the CD25 MicroBead Kit (Miltenyi Biotec), and CD4^+^ T cells were subsequently isolated with the MagniSort™ Mouse CD4 T cell Enrichment Kit (Thermo Fisher). The T cells were then labeled with 5 μM CellTrace™ Violet (Thermo Fisher) and cocultured for 4 days with either BMDCs or SPDCs at a ratio of 5:3 (50,000 T cells:30,000 DCs) with or without 2 ng/ml human TGF-β (R&D Systems) in the presence of 50 U of human IL-2 (PeproTech).

### Adoptive transfer of cells

Isolated CD8^+^ OT-I T cells were labeled with 5 μM CellTrace™ Violet (Thermo Fisher). A total of 5 × 10^5^ OT-I cells were administered to each mouse via the tail vein.

### Flow cytometry, antibodies, and staining

For flow cytometry analysis, single-cell suspensions were stained with fluorochrome-conjugated antibodies. Cells were stained for CD3e (145-2C11), NK1.1 (PK136), CD19 (MB19-1), CD11c (N418), CD11b (M1/70), TER-119 (TER-119), and Ly-6G and Ly-6C (RB6-8C5) as lineage markers or for Flt3 (A2F10), c-kit (2B8), Sca-1 (E13-161.7), CD16/32, CD127 (A7R34), CD49b (DX-5), NK1.1 (PK136), CD8a (53-6.7), CD19, Thy1.2 (53-2.1), CD4 (RM4-5), I-A/E (M5/114.15.2), CD11b (M1/70) and CD11c (N418) in the presence of an anti-NP monoclonal antibody (VL-4; BioXCell) conjugated to Alexa Fluor 647 (Molecular Probes) in our laboratory. BMDCs were costained with PE-conjugated antibodies against CD80 (16-10A1), CD86 (GL1), PD-L1 (MIH5), B7-H4 (188), PVR (3F1), H-2K^b^ (AF6-88.5), I-A/I-E (M5/11415.2) and H-2K^b^ bound to SIINFEKL (25-D1.16) or corresponding isotype antibodies to detect various accessory molecules on BMDCs. To detect intracellular cytokines expressed in CD8^+^ T cells, CD8^+^ T cells cocultured with DCs were incubated with 0.2 μg/ml LCMV GP_33-41_ peptide in the presence of GolgiPlug GolgiStop™ (BD Biosciences), and intracellular staining for cytokines was performed by using anti-IFN-γ (XMG1.2), anti-IL-2 (JES6-5H4) and anti-TNF-α (MP6-XT22) antibodies. For Treg cell staining, cells were fixed, permeabilized with a Foxp3 Transcription Factor Staining Buffer Set (Thermo Fisher) and incubated with an anti-Foxp3 (FJK16s) antibody to evaluate the intracellular expression of Foxp3 in Treg cells. All antibodies were purchased from BD Biosciences except for the anti-CD127, anti-CD11b, anti-F4/80, anti-I-A/I-E, anti-PD-L1, and anti-Foxp3 antibodies, biotin-conjugated antibodies (Thermo Fisher), and the anti-PD-1 antibody (BioLegend). The LIVE/DEAD™ Fixable Dead Cell Stain Kit (Invitrogen) was used to identify live cell population in most staining procedures. All stained samples were analyzed using a FACSCanto II (BD Biosciences) and Cytoflex LX (Beckman Coulter).

### Flow cytometry data analysis

All the data saved as FCS files were assessed using FlowJo software (TreeStar). tSNE clustering was applied with the Barnes-Hut implementation algorithm using the following parameter: perplexity = 30.

### Quantitative RT‒PCR

RNA was isolated from BMDCs using the RNeasy Mini Kit (QIAGEN), and cDNA was synthesized using SuperScript® II Reverse Transcriptase according to the manufacturer’s protocol (Invitrogen). The expression levels of individual genes were measured by quantitative PCR (qPCR) on a CFX96 real-time PCR detection system (Bio-Rad) with the following gene-specific primers: *Ido* F: GTACATCACCATGGCGTATG and R: CGAGGAAGAAGCCCTTGTC; *Tgf-b* F: TGATACGCCTGAGTGGCTGTC and R: TTGATTTCCACGTGGAGTTTG; *Il-10* F: CTTACTGACTGGCATGAGGATCA and R: GCAGCTCTAGGAGCATGTGG; and *Gapdh* F: GGCAAATTCAACGGCACAGTCAAG and R: TCGCTCCTGGAAGATGGTGATGG. Relative mRNA expression levels were normalized to the *Gapdh* level. qPCR was performed as follows: initial denaturation of the template for 3 minutes at 95 °C followed by 45 cycles of 10 seconds at 94 °C, 20 seconds at 60 °C, and 30 seconds at 72 °C. The final reaction volume was 20 μl, and SYBR Green I (QIAGEN) was used to detect PCR products according to the manufacturer’s recommendation.

### ELISA

To measure cytokine production, cell culture supernatants were collected and stored at −70 °C. The levels of cytokines were analyzed with sandwich ELISA kits (Thermo Fisher).

### Multiple bead-based immunoassay

To measure the production of multiple cytokines, cell culture supernatants were collected, and the amount of each cytokine in the supernatant was analyzed using the Mouse Inflammation Panel (13-plex) and Murine Th1/Th2 Panel (8-plex) of LEGENDplex™ (BioLegend). The concentration of each cytokine was analyzed using flow cytometry (CytoFLEX; Beckman Coulter) and LEGENDplex™ software (BioLegend).

### Immunoblot analysis

On Day 9 of BMDC differentiation, nonadherent cells were harvested, reseeded in 6-well plates at a density of 1 × 10^6^ cells/ml and starved for 5 hours in serum-free RPMI. After serum starvation, the cells were stimulated with LPS (1 μg/ml) for 0 to 2 hours and then lysed in 150 μl of lysis buffer containing RIPA buffer (Thermo Fisher Scientific), a phosphatase inhibitor, a protease inhibitor, and an EDTA inhibitor for 40 minutes. Whole-cell lysates were resolved on 2% SDS-polyacrylamide gels and then transferred to nitrocellulose membranes for 1.5 hours at 90 V. The membranes were blocked in 5% skim milk and incubated with primary antibodies for 18 hours at 4 °C, followed by incubation with HRP-conjugated secondary antibodies for 1.5 hours at room temperature. Target proteins were labeled with primary antibodies against total MAPKs and IκB-α and polyclonal antibodies against p-p38, p-ERK1/2, p-JNK, and p-IκB-α (all antibodies from Cell Signaling Technology were diluted 1:1000 in the blocking solution). The results were visualized by using the ECL Advance Western Blotting Detection Kit (GE Healthcare).

### Treatment with pharmacological inhibitors

On Day 9 of BMDC culture, BMDCs were treated with SB203580, U0126, or SP600125 (all inhibitors were from Santa Cruz and were reconstituted in sterile 0.05% DMSO) for 1 hour prior to LPS treatment (500 ng/ml). After 18 hours of LPS treatment, the culture medium was collected and frozen at −70 °C. The level of IL-10 was estimated by sandwich ELISA.

### PD-L1 blockade

For blockade of PD-L1 expressed on CD11b^+^ BMDCs or SPDCs, isolated DCs were incubated with 50 μg/ml anti-PD-L1 antibody (10 F.9G2; BioXCell) or rat IgG2b isotype control antibody (LTF-2; BioXCell) for 2 hours in vitro and washed twice with PBS before coculture with CD8^+^ T cells.

### Statistical analysis

The statistical significance of differences among groups was analyzed by performing multiple-comparison analysis with one-way or two-way ANOVA using PRISM software version 7.0 (GraphPad). The *p* values in the figures indicate the following: *P < 0.05; **P < 0.01; and ***P < 0.001.

## Results

### Chronic infection with LCMV CL13 altered the frequency and number of multiple progenitor cells and lineage-differentiated cells

**Fig. 1:**
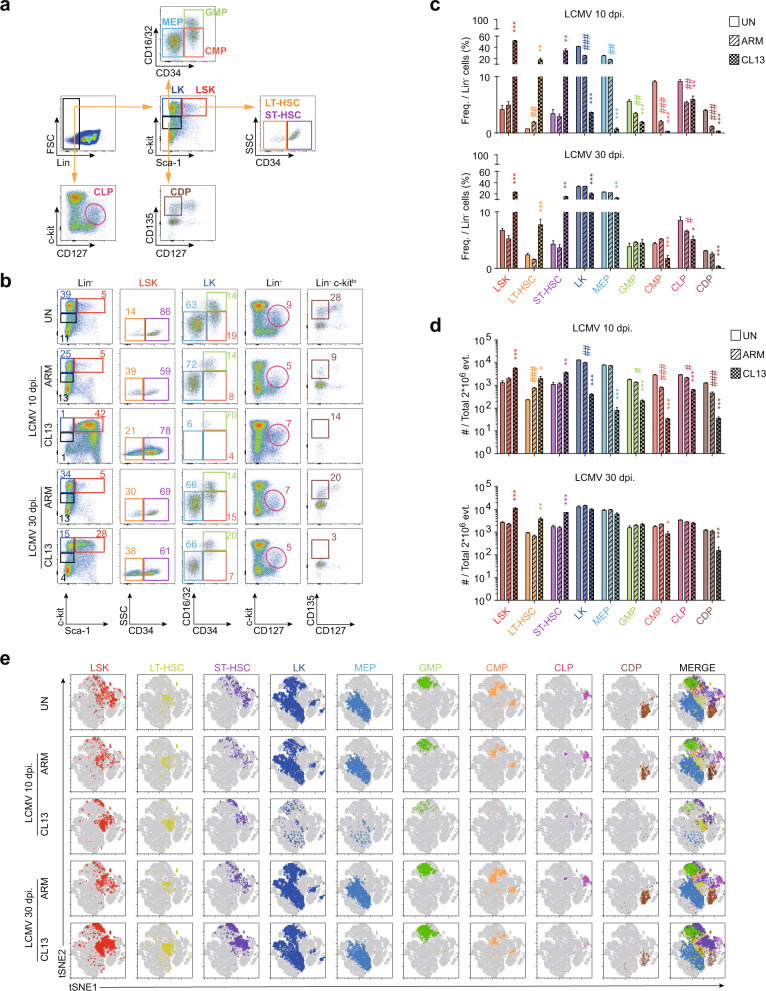
HSCs, committed progenitor cell populations in the BM, are altered upon chronic LCMV CL13 infection. **a** Gating strategies for identifying different populations in the BM by surface marker expression: LSK cells (Lin^−^, Sca-1^+^, c-kit^+^), LK cells (Lin^−^, Sca-1^−^, c-kit^+^), LT HSCs (Lin^−^, Sca-1^+^, c-kit^+^, CD135^−^, CD34^−^), ST HSCs (Lin^−^, Sca-1^+^, c-kit^+^, CD135^−^, CD34^+^), MEPs (Lin^−^, Sca-1^−^, c-kit^+^, CD16/32^low^, CD34^−^), GMPs (Lin^−^, Sca-1^−^, c-kit^+^, CD16/32^high^, CD34^+^), CMPs (Lin^−^, Sca-1^−^, c-kit^+^, CD16/32^low^, CD34^+^), CLPs (Lin^−^, c-kit^low^, CD135^+^, CD127^+^), and CDPs (Lin^−^, Sca-1^−^, c-kit^low^, CD135/CD115^+^, CD127^−^). **b** Each HSC and progenitor population in the BM of uninfected or LCMV-infected mice (10 or 30 days postinfection) was identified by gating on surface markers. **c** The frequency of each HSC and progenitor cell in the lineage marker-negative populations between the uninfected and LCMV-infected groups is summarized in a bar plot. Graphs for 10 days post-LCMV infection (early chronic phase) and 30 days post-LCMV infection (late chronic phase) are presented. **d** The absolute numbers of HSCs and progenitor cells in two million live BM cells were analyzed. **e** tSNE visualization of HSC and progenitor cell populations in the BM. Each population was visualized at distinct locations on the tSNE map, and clustering was applied with the Barnes-Hut implementation algorithm using the following parameter: perplexity = 30. The bar graphs show the means ± SDs (5 mice in each group). Each experiment was repeated 3 times. The *p* values in the figures indicate the following: *P < 0.05; **P < 0.01; **P < 0.01; ***P < 0.001.

To understand the susceptibility of stem cells and progenitor cells to LCMV infection, we first analyzed the frequency of HSCs and committed progenitors in the BM of naive and virus-infected mice. Each HSC or progenitor subset was distinguished by the expression of surface markers as described in the gating strategy for the lineage marker-negative population (Fig. 1a)^21–23^. The frequencies and numbers of lineage^-^ Sca-1^+^ c-kit^+^ (LSK) stem cell populations, including long-term (LT) and short-term (ST) HSCs, were extremely high after 10 days of LCMV CL13 infection, whereas the frequencies and numbers of other lineage-negative progenitor cells subordinate to HSCs in the hematopoietic hierarchy, such as megakaryocyte-erythroid progenitors (MEPs), granulocyte-macrophage progenitors (GMPs), common myeloid progenitors (CMPs), common lymphoid progenitors (CLPs) and common DC progenitors (CDPs), were very low (Fig. 1b–d). These results indicated the possibility that a drastic increase in the viral load or a maintained high viral load modulated hematopoiesis in the BM. To confirm this, we additionally analyzed HSC and progenitor populations in the BM in early phases of LCMV infection, such as on Days 3 and 5 postinfection. At these timepoints, most lineage-negative populations exhibited the LSK phenotype regardless of acute LCMV ARM or chronic LCMV CL13, and the overall composition of populations in the BM was similar to that on Day 10 of CL13 infection (Supplementary Fig. 1). Moreover, we analyzed the compositions of HSCs and progenitors in the late phase of LCMV infection. Interestingly, the frequencies and absolute numbers of CMP and CDP populations directly related to DC differentiation had still not recovered on Day 30 after CL13 infection, whereas those of other progenitors had mostly recovered (Fig. 1b–d). In addition, we visualized the indicated HSC and progenitor cell populations with a t-distributed stochastic neighbor embedding (tSNE) plot using the hematopoietic markers described above (Fig. 1e). Each population was present at a distinct location, and the marked population intensity was consistent with the earlier data in Fig. 1b.

**Fig. 2:**
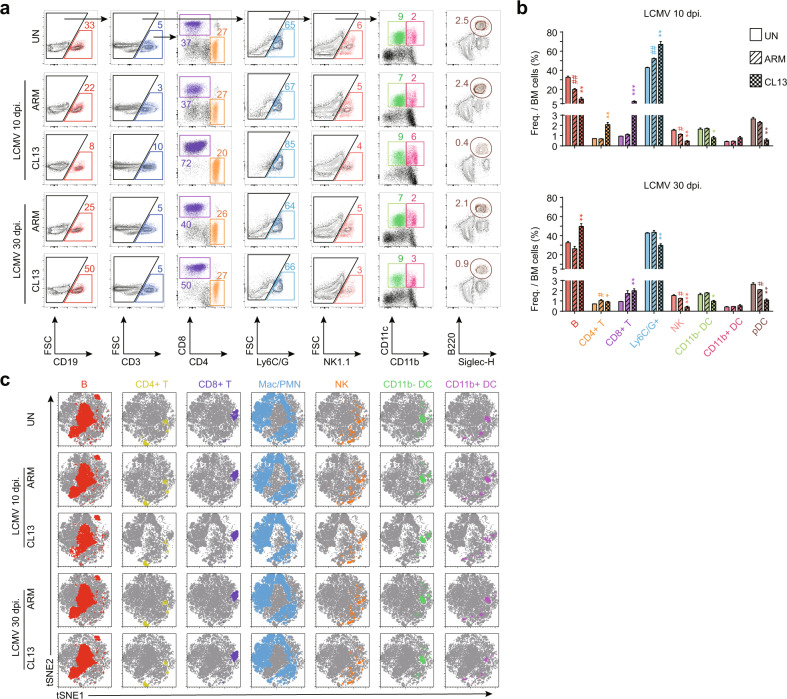
Lineage-differentiated mature cell populations in the BM are altered upon chronic LCMV CL13 infection. **a** Differentiated immune cells in the BM of naive or LCMV-infected mice (10 or 30 days postinfection) were analyzed with lineage markers: B cells (CD19^+^), NK cells (NK1.1^+^), CD4^+^ T cells (CD3/Thy1.2^+^, CD4^+^), CD8^+^ T cells (CD3/Thy1.2^+^, CD8^+^), macrophages or PMN cells (F4/80^+^ or Ly6C/G^+^), myeloid DCs (non-T cells/B cells/NK cells/macrophages/PMN cells, CD11c^+^, MHCII^high^), and pDCs (CD11c^int^, B220^+^, Siglec-H^+^). **b** The frequencies of lineage-differentiated immune cell populations in the BM were analyzed. **c** tSNE visualization of each lineage-differentiated immune cell population in the BM. The bar graphs show the means ± SDs (5 mice in each group). Each experiment was repeated 3 times. The *p* values in the figures indicate the following: *P < 0.05; **P < 0.01; **P < 0.01; ***P < 0.001.

In addition to the changes in HSCs and progenitors, we identified quantitative changes in lineage-differentiated and mature typical immune cells in the BM during LCMV infection. Immune cells such as B, T, NK, polymorphonuclear (PMN) and dendritic cells in the BM were identified by their lineage markers (Fig. 2a). These lineage-differentiated immune cells also showed dramatic changes in both frequency and number. The numbers and percentages of myeloid-lineage and NK cells were decreased, and this pattern was maintained in the BM (Fig. 2b) and spleen (data not shown) throughout chronic viral infection. We additionally visualized each immune cell population on the tSNE map; however, it was difficult to find a distinct population intensity difference between groups similar to that observed in the map of HSCs and progenitor cells (Fig. 2c). Taken together, these results indicated that chronic LCMV infection affected a fundamental step of hematopoiesis, restraining HSC differentiation into progenitor cells. Furthermore, the populations showing the most consistent suppression of differentiation in the BM during CL13 infection were CMPs and CDPs, which are in the stages of myeloid-lineage cell differentiation. This persistent altered differentiation in the myeloid lineage during CL13 infection could affect overall immune cell development and responses since myeloid cells not only contribute to innate immune responses but also play a role as APCs.

### BM-derived CD11b/c^+^ myeloid DCs in chronically infected mice showed a less immunogenic phenotype

**Fig. 3:**
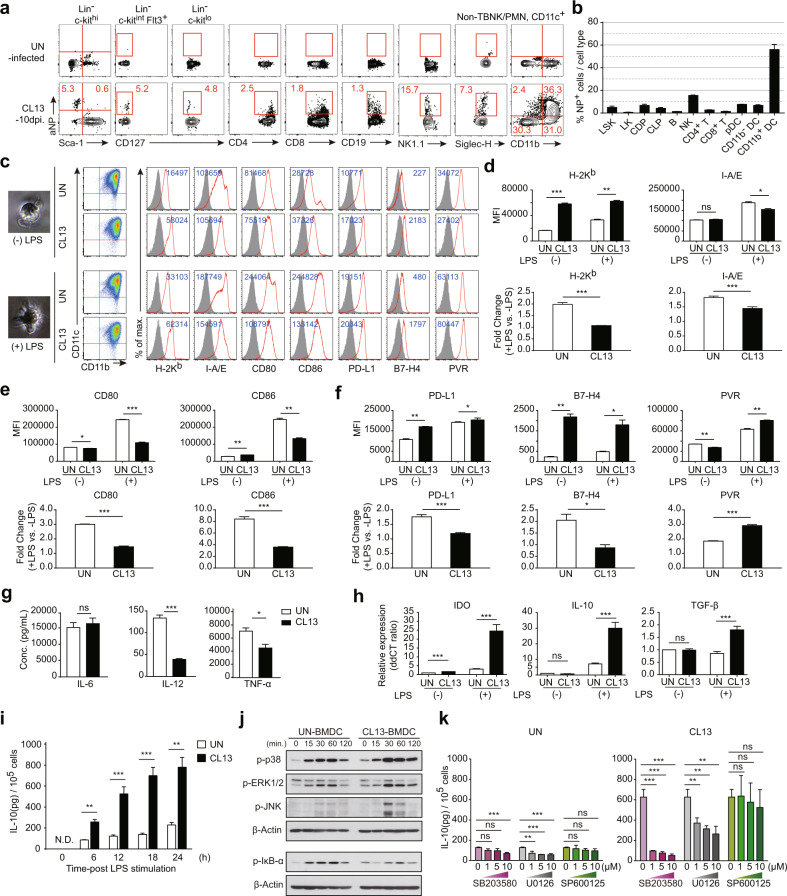
CD11b^+^ myeloid DCs differentiated during chronic CL13 infection exhibit relatively weakly immunogenic phenotypes. **a** LCMV-infected populations were stained for NP, and the frequency of NP^+^ cells was analyzed by flow cytometry. The percentage of NP^+^ cells among each HSC, progenitor and lineage cell type is shown in FACS plots. **b** The proportion of the NP^+^ population in each stem cell, progenitor and lineage cell population is presented in a bar graph. **c** Phenotypic and morphological analyses of BMDCs differentiated from uninfected or CL13-infected mice were conducted. The MFI is shown in the plot. Bar graphs presenting the MFI values of **d** MHC, **e** costimulatory and **f** inhibitory molecules on the BMDC surface. The MFI fold-change of each molecule upon LPS treatment is also displayed. **g** The total amount of each cytokine secreted by BMDCs was measured by ELISA. **h** RNA was isolated from BMDCs, and the relative expression levels of IDO, IL-10, and TGF-β were evaluated. **i** Kinetics of IL-10 expression in BMDCs from naive or CL13-infected mice at the protein level. BMDCs were treated with LPS (500 ng/ml) for 0, 6, 12, 18, or 24 hours. The IL-10 levels in the culture medium of BMDCs from uninfected or CL13-infected mice were measured by sandwich ELISA. **j** MAPK signaling activation in BMDCs was analyzed by western blot analysis. Uninfected and CL13-infected BMDCs were treated with LPS for 0, 15, 30, 60, or 120 minutes. All cell lysates were subjected to SDS‒PAGE, and then immunoblotting was conducted using antibodies specific for p-p38, p-ERK, p-JNK and p-IκB-α. **k** BMDCs from uninfected and CL13-infected mice were treated with a pharmacological inhibitor of p38 (SB203580), ERK (U0126), or JNK (SP600125) for 1 hour and then treated with LPS for 18 hours. The IL-10 levels in the cell culture supernatant were quantitated by ELISA. The bar graphs show the means ± SDs (*n* = 3 samples). Each experiment was repeated 3 times. The *p* values in the figures indicate the following: *P < 0.05; **P < 0.01; **P < 0.01; ***P < 0.001.

Hematopoietic progenitor cells act as a reservoir in persistent HIV infection^15,24^; thus, we investigated whether BM can serve as a long-term viral reservoir in chronic LCMV infection. We determined the viral titer in the BM via a plaque formation assay. LCMV CL13 viral titers were detectable in BM cells at 10 and 20 days post-CL13 infection (Supplementary Fig. 2a) as well as on Day 30 of CL13 infection^25^. Next, we examined LCMV-infected populations in the BM during LCMV CL13 infection by staining for intracellular LCMV NP. We were unable to detect any NP^+^ LT or ST HSCs, whereas CDPs, CLPs and mature-lineage cell types were found to be NP^+^. Notably, after LCMV CL13 infection, approximately 40% of CD11c^+^ cells were NP^+^ cells, almost all of which were also CD11b^+^ (Fig. 3a, b), suggesting that LCMV CL13 mainly infects CD11b/c^+^ DC subsets despite their sparsity (Fig. 2a, b)^26^. These LCMV NP^+^ CD11b^+^ DCs were also detectable in the late chronic phase (Supplementary Fig. 2b).

Given these findings, we further focused on myeloid cell differentiation and the CD11c^+^ population, especially CD11b^+^ myeloid DCs. To examine how infected CD11b^+^ myeloid DCs establish immune responses during chronic LCMV infection, we generated BM-derived myeloid DCs (BMDCs) in vitro^27,28^. BM cells were isolated from naive (uninfected, UN) and LCMV-infected (ARM or CL13) mice and differentiated in the presence of murine GM-CSF in vitro. Consistent with our previous data showing that CL13 infection very strongly restrained immune cell differentiation (Fig. 1), the frequency and number of CD11b/c^+^ cells in the context of CL13-infected BMDC differentiation were much lower than those in uninfected or ARM-infected conditions (Supplementary Fig. 2c). On Days 9 and 10 of BMDC culture, we harvested immature BMDCs in suspension and observed their morphology and CD11b/c levels. Harvested BMDCs from either naive or LCMV-infected mice were CD11b/c^+^ and showed a dendritic morphology (Fig. 3c). To identify factors that could contribute to T cell dysfunction or immune suppression by CL13-infected myeloid DCs, we conducted phenotypic and functional analyses of BMDCs, focusing especially on immunosuppressive factors. Using flow cytometry, we first analyzed the expression of surface membrane proteins such as MHCs (Fig. 3d), costimulatory molecules (Fig. 3e), and inhibitory molecules (Fig. 3f) on BMDCs under steady-state conditions (without LPS treatment, LPS-). Differentiated BMDCs from CL13-infected mice presented higher expression of H-2K^b^, CD86, PD-L1, and B7-H4 but lower levels of CD80 and PVR than UN-BMDCs. Although differentiated BMDCs from ARM-infected mice presented different expression patterns, showing slightly higher expression of H-2K^b^, I-A/E, CD80, CD86 and PVR, the expression pattern and mean fluorescence intensity (MFI) of these BMDCs were similar to those of naive BMDCs rather than those of CL13-BMDCs (Supplementary Fig. 2d–g). These phenotypic differences among UN-BMDCs and ARM- and CL13-infected BMDCs likely resulted from a non- or preactivated state and alteration of HSCs by existing viral antigens. To demonstrate that the phenotypic differences may have been constantly maintained during overall differentiation and be reflected in different kinetics of DC activation, we performed a time course phenotypic analysis of BMDCs during differentiation and induced fully activated or mature BMDCs by treating cells with another stimulus, LPS. On Day 4, an early timepoint in BMDC differentiation, the percentage of the CD11b/c^+^ population in CL13-infected BMDCs was only half of that in naive BMDCs, which was consistent with our earlier data indicating restrained hematopoiesis in chronic viral infection. In addition, the comparative expression patterns of surface molecules among UN-BMDCs and ARM-, and CL13-infected BMDCs were similar to those on Day 10 (Supplementary Fig. 2h, i). Next, we treated BMDCs with LPS to fully activate and mature the BMDCs. Interestingly, compared to naive BMDCs, CL13-infected BMDCs did not show drastic changes in the expression of surface molecules other than PVR before and after LPS treatment; this resulted in higher expression of inhibitory molecules but lower expression of costimulatory molecules in CL13-infected BMDCs than in naive BMDCs. Differences in the activation and sensitivity to stimuli between UN-BMDCs and CL13-infected BMDCs were indicated by the MFI fold-change of each surface molecule (Fig. 3d–f). Taken together, the results indicated that since BMDCs generated during chronic viral infection were already activated and since maturation was induced by existing viral antigens, the cells were unable to be drastically more activated than those in uninfected conditions, and they seemed to be less sensitive to other stimuli.

Next, in addition to assessing the expression of surface molecules, we evaluated the expression of inflammatory cytokines and immunoregulatory molecules on BMDCs. Representative inflammatory cytokines expressed by DCs, such as IL-6, IL-12 and TNF-α, were quantified by ELISA. Compared to that in naive BMDCs, the expression of IL-12 and TNF-α in CL13-BMDCs was reduced, while only that of IL-12 was slightly decreased in ARM-infected BMDCs (Fig. 3g and Supplementary Fig. 2j). We also estimated the mRNA expression of several key immunosuppressive factors, such as IL-10, TGF-β, and indoleamine 2,3-dioxygenase (IDO), in BMDCs by qPCR. Upregulation of IDO expression can alter the function of APCs and change the milieu from immunogenic to tolerogenic^29–33^. In addition, activation of suppressive Treg cells constitutes one of the major pathways through which IDO can affect T cell immune responses^29^. TGF-β is important for the maintenance and survival of Treg cells^34^, and IL-10 serves as a key cytokine for Treg cell induction^35,36^, which can also suppress the cytokine production and proliferation of CD4^+^ and CD8^+^ T cells and alter the functions of APCs, such as DCs and macrophages^37^. Using quantitative RT‒PCR, we confirmed that BMDCs from CL13-infected mice showed notably elevated expression of IDO, IL-10, and TGF-β, which can promote immune suppression (Fig. 3h)^38^. We further focused on IL-10, an immunosuppressive cytokine that functions as a master regulator of immunity in various infections^39^ and is an important factor contributing to T cell exhaustion during chronic LCMV infection^40^. Upon LPS stimulation, the amount of IL-10 secreted by CL13-infected BMDCs was much higher than that secreted by BMDCs from naive mice, which was consistent with the previous mRNA expression data obtained by quantitative RT‒PCR (Fig. 3i). The expression of IL-10 in DCs is largely related to the activation of MAPK and NF-κB^41–45^; thus, to test whether this extreme increase in the IL-10 level in DCs from CL13-infected mice was associated with the classic MAPK signaling cascade, we performed a pharmacological inhibitor treatment assay. First, we measured the expression levels of the phosphorylated forms of the MAPK p38, ERK, and JNK by western blot analysis. In addition, the phosphorylation and degradation of IκB-α, a component of the upstream NF-κB signal transduction cascade, were examined by western blotting. BMDCs from CL13-infected mice expressed higher levels of the phosphorylated forms of the three MAPKs than BMDCs from naive mice, but CL13-infected BMDCs exhibited less phosphorylation and degradation of IκB-α. These changes were significant at 30 minutes after LPS treatment and appeared to persist until 2 hours after treatment (Fig. 3j). This finding suggests that the MAPK (p38, ERK, and JNK) and NF-κB signaling pathways may contribute to IL-10 production in BMDCs from CL13-infected mice. To further clarify whether the different expression patterns of MAPKs observed by western blot analysis actually caused the differences in IL-10 levels between UN-BMDCs and CL13-infected BMDCs, we administered pharmacological inhibitors of the three MAPKs (SB203580 for p38, U0126 for ERK, and SP600125 for JNK) in combination with LPS to BMDCs and then measured IL-10 production. Treatment with the p38 or ERK inhibitor abated IL-10 production in BMDCs from either naive or CL13-infected mice, whereas inhibition of JNK signaling had no effect. Notably, the p38 inhibitor markedly decreased the IL-10 level in BMDCs from CL13-infected mice to a level similar to that in BMDCs from naive mice (Fig. 3k). These findings implied that IL-10 production in BMDCs from CL13-infected mice was increased through activation of the MAPK signaling pathway, especially activation of p38 MAPK, and that IL-10 in DCs may be an immunosuppressive signal in T cell activation or functionality.

These results suggested that CD11b^+^ myeloid DCs differentiated during CL13 infection exhibited reduced sensitivity to stimuli and reduced immunogenicity, as indicated by their reduced secretion of inflammatory cytokines and increased production of immunoregulatory factors.

### CD11b/c^+^ DCs contributed to functional alterations in virus-specific CD8^+^ T cells and the induction of Foxp3^+^ Treg cells during chronic viral infection

**Fig. 4:**
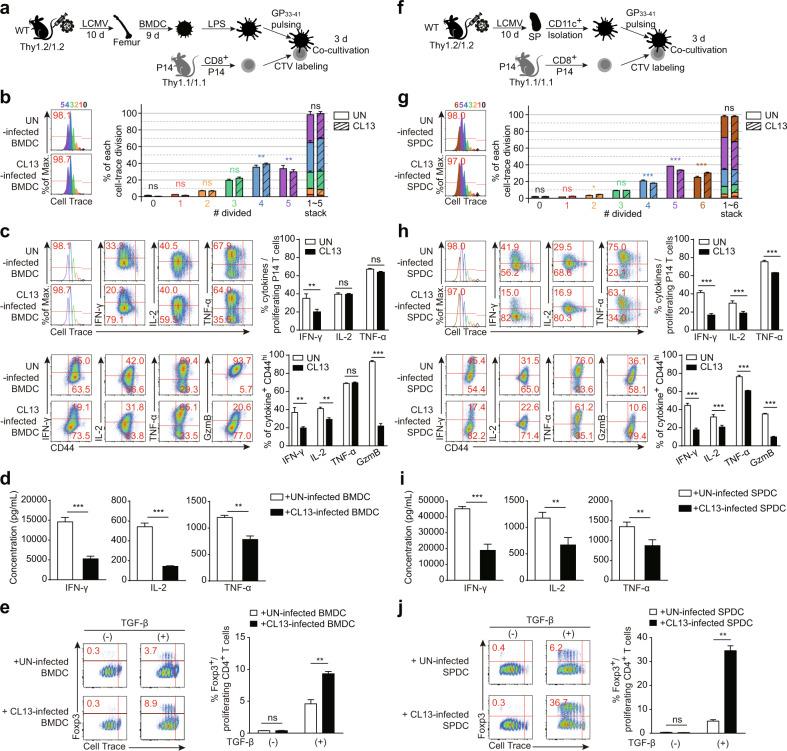
LCMV CL13-infected DCs induce functionally defective CD8^+^ T cells and more Foxp3^+^ Treg cells. **a** Experimental scheme and strategy for the T cell functional assay using BMDCs in vitro. BM cells were isolated from naive and CL13-infected mice (CL13 10 dpi.) and differentiated in the presence of GM-CSF in vitro. Mature BMDCs treated with LPS were cocultured with CellTrace-labeled LCMV-specific CD8^+^ P14 T cells for 3 days. **b** The proliferation and division of P14 T cells cultured with UN-BMDCs or CL13-infected BMDCs were analyzed by CellTrace dye staining. The bar graph presents the percentage of the cell populations in each division number. **c** Potential cytokine secretion by P14 T cells was analyzed by intracellular cytokine staining and CellTrace dye staining. The kinetics of effector cytokine expression during P14 T cell proliferation were analyzed (top two FACS plots and bar graph). The expression levels of IFN-γ, IL-2, TNF-α and granzyme B in activated CD44^hi^ proliferating P14 T cells were investigated (bottom two FACS plots and bar graph). **d** The total amount of each cytokine expressed in the supernatant of P14-BMDC cocultures was measured by a multiplex bead assay. **e** CellTrace-labeled CD4^+^ T cells were cocultured with UN-BMDCs or CL13-infected BMDCs for 4 days with or without exogenous TGF-β, and the induction of Foxp3^+^ Treg cells was compared. **f** Experimental scheme and strategy for the T cell functional assay using ex vivo SPDCs. CD11b^+^ SPDCs were isolated from the spleen of naive or CL13-infected (10 days postinfection) mice and cocultured with CellTrace-labeled LCMV-specific CD8^+^ P14 T cells for 3 days under GP_33-41_ peptide stimulation. **g** The proliferation and division of P14 T cells cultured with SPDCs from uninfected or CL13-infected mice were analyzed by CellTrace dye staining. The bar graph presents the percentage of the cell populations in each division number. **h** The cytokine-secreting potential of P14 T cells cocultured with SPDCs was analyzed. The kinetics of effector cytokine expression during P14 T cell proliferation were analyzed (top two FACS plots and bar graph). The expression levels of IFN-γ, IL-2, TNF-α and granzyme B in activated CD44^hi^ proliferating P14 T cells were investigated (bottom two FACS plots and bar graph). **i** The total amount of each cytokine expressed in the supernatant of P14-SPDC cocultures was measured by a multiplex bead assay. **j** Myeloid SPDCs were cocultured with CellTrace-labeled CD4^+^ T cells for 4 days with or without exogenous TGF-β, and the induction of Foxp3^+^ Treg cells was compared. The bar graphs show the means ± SDs (6 mice in each group). Each experiment was repeated twice. The *p* values in the figures indicate the following: *P < 0.05; **P < 0.01; **P < 0.01; ***P < 0.001.

According to the data shown above, we hypothesized that at the system level, BMDCs generated during chronic LCMV infection could contribute to immune suppression or T cell dysfunction. Therefore, to investigate the function of DCs from naive mice and chronically infected mice in priming the effector T cell immune response, we cocultured T cells with BMDCs in vitro^46^. Although BMDCs from CL13-infected mice were already mature and loaded with antigens, naive BMDCs were completely unable to prime T cells without antigen pulsing. LPS-stimulated fully mature BMDCs from naive mice induced T cell proliferation in an antigen-independent manner but to a negligible extent. Therefore, the minimal antigen dose that enabled T cell priming (the threshold dose) had to be applied, and that amount of GP_33-41_ peptide stimulation did not induce any evident defects in proliferation (Supplementary Fig. 3)^47^. To assess functional alterations in LCMV-specific effector T cells, the proliferation and division of P14 cells were examined by CellTrace dye staining. CellTrace-labeled P14 CD8^+^ T cells were cocultured with BMDCs from either naive or CL13-infected mice in the presence of the GP_33-41_ peptide for 3 days (Fig. 4a), and the proliferation and cytokine production of the antigen-specific T cells were then analyzed. There were no significant differences in frequency or number among the dividing or proliferated cells (Fig. 4b). We also assessed the potential and kinetics of effector cytokine expression during T cell proliferation via intracellular cytokine staining. Evaluation of the expression level of each effector cytokine upon the CellTrace axis revealed that IFN-γ expression was reduced in proliferating P14 T cells primed with CL13-BMDCs. We further assessed the expression levels of IFN-γ, IL-2, TNF-α and granzyme B (GzmB) in the activated CD44^hi^ CD8^+^ T cell population. The expression of IFN-γ, IL-2 and GzmB was significantly reduced in activated P14 cells primed with CL13-BMDCs (Fig. 4c). In addition to testing the potential for cytokine expression by intracellular cytokine staining, we evaluated the total amounts of effector cytokines present in the supernatant following coculture. At the protein level, secretion of all three effector cytokines was reduced in P14 T cells cocultured with CL13-BMDCs (Fig. 4d).

Given the decreased efficiency of BMDCs from CL13-infected mice in inducing the production of T helper 1 effector cytokines and high levels of IDO, IL-10 and TGF-β (Fig. 3h), we next determined whether these myeloid DCs could promote the induction of Foxp3^+^ Treg cells by coculturing BMDCs with CD4^+^ T cells. CD4^+^ CD25^-^ T cells isolated from the spleen of naive mice were cocultured with BMDCs for 4 days in the presence or absence of TGF-β. Interestingly, CL13-infected BMDCs induced 2.6-fold greater Foxp3^+^ Treg cell expansion than did UN-BMDCs or ARM-infected BMDCs in the presence of TGF-β (Fig. 4e and Supplementary Fig. 4d).

However, P14 T cells primed with ARM-infected BMDCs proliferated equally to P14 T cells primed with naive BMDCs, and a defect in effector cytokine expression was not observed, but the expression of TNF-α was slightly upregulated (Supplementary Fig. 4a–c). Moreover, ARM-infected BMDCs could not expand Foxp3^+^ Treg cells as much as CL13-infected BMDCs did (Supplementary Fig. 4d).

We next extended our findings to mouse-derived myeloid SPDCs by generating ex vivo experimental models. First, phenotypic analysis of splenic CD11b^+^ myeloid DCs was performed on Day 10 after LCMV infection. The expression of surface molecules such as H-2K^b^, CD80, CD86, PD-L1 and PVR on splenic CD11b^+^ DCs was upregulated in CL13-infected mice compared to naive mice. The expression of PD-L1 and PVR on CL13-infected SPDCs was particularly increased by 2.0-fold and 22.0-fold, respectively (Supplementary Fig. 2k, l). These comparative expression patterns of surface molecules between naive and CL13-infected mice were preserved on Day 30 after LCMV infection; however, the absolute expression of CD80 and CD86 was lower, whereas the expression of PD-L1 was higher, at 30 days post-CL13 infection than at 10 days post-CL13 infection (data not shown). To investigate the alterations in CD8^+^ T cell functionality caused by splenic CD11b^*+*^ DCs, we primarily removed CD19^+^, CD49b^+^, and CD90.2^+^ populations with a microbead cocktail and blocked CD16/32 with a purified anti-CD16/32 antibody. In sequence, we isolated the CD11c^+^ DC population and found that 90% of these CD11c^+^ cells were CD11b^+^ myeloid cells. Then, these SPDCs isolated from naive or CL13-infected mice were cocultured with either P14 CD8^+^ T cells or CD4^+^ T cells in vitro (Fig. 4f). The results obtained for ex vivo myeloid SPDCs were similar to those for in vitro-differentiated BMDCs. Under GP_33-41_ peptide stimulation, CD8^+^ T cells primed with SPDCs from naive or CL13-infected mice proliferated at the same rate overall (Fig. 4g), whereas P14 T cells primed with SPDCs from CL13-infected mice expressed 2.8-fold less IFN-γ and 1.2-fold less IL-2 and TNF-α. The expression of GzmB was also decreased by 6.0-fold in P14 T cells primed with SPDCs from CL13-infected mice (Fig. 4h). Similarly, the total secreted amounts of these cytokines were diminished when P14 T cells were primed with SPDCs generated from CL13-infected mice (Fig. 4i). Similar to the case with P14 T cell and ARM-infected BMDC coculture, the proliferation of P14 T cells primed with SPDCs from ARM-infected mice was equal to that of the control group (Supplementary Fig. 4e), and effector cytokine expression in CD8^+^ T cells was not suppressed (Supplementary Fig. 4f, g). In the assessment of the capacity to induce Foxp3^+^ Treg cells, SPDCs from CL13-infected mice produced 6.0-fold more Treg cell expansion than did SPDCs from uninfected or ARM-infected mice (Fig. 4j and Supplementary Fig. 4h). Additionally, to manipulate and expand CD11b^+^ DCs selectively in vivo, we subcutaneously injected B16F10 melanoma cells engineered to secrete GM-CSF^48^ into the flank of mice 9 days before LCMV infection (Supplementary Fig. 5a). Injection of this engineered cell line selectively expanded the CD11b/c^+^ myeloid SPDC population (Supplementary Fig. 5b). Expanded CD11b^+^ SPDCs from this system also could not efficiently prime CD8^+^ T cells to produce effector cytokines (Supplementary Fig. 5c). In addition, more Foxp3^+^ Treg cells were induced when CD4^+^ T cells were cocultured with selectively expanded CD11b^+^ SPDCs from CL13-infected mice (Supplementary Fig. 5d).

In recent decades, to restore the impaired function of immune cells and enhance immune responses, studies and clinical trials have been widely conducted on immune checkpoint blockade^49,50^. The PD-1 and PD-L1 axis between T cells and APCs or T cells and tumor cells contributes to the downregulation of immunity; thus, inhibition of signaling between PD-1 and PD-L1 could enhance T cell functionality and improve the treatment of disease^51–54^. Since we found that the expression of PD-L1 was upregulated on CD11b^+^ myeloid DCs during chronic LCMV infection, we further investigated whether blockade of the PD-1/PD-L1 interaction during in vitro stimulation could rescue the deficient cytokine production in CD8^+^ T cells. The expression of PD-L1 on BMDCs was blocked by treatment with a purified anti-PD-L1 monoclonal antibody for 2 hours (Supplementary Fig. 6a). Then, the treated BMDCs were cocultured with P14 T cells as previously described. The expression of PD-1, a receptor of PD-L1, was slightly elevated on P14 T cells cocultured with CL13-infected BMDCs. Blockade of PD-L1 on CL13-infected BMDCs did not affect the proliferation of P14 T cells and could not restore the functionality of these CD8^+^ T cells with regard to effector cytokine expression (Supplementary Fig. 6b). The frequency of effector cytokine-expressing activated T cells was not altered by blockade of PD-L1 in either the UN or CL13-infected group (Supplementary Fig. 6c). In accordance with the intracellular cytokine staining data, the total amount of each effector cytokine in the supernatant of P14 T cell and CL13-infected BMDC cultures was not increased by blocking PD-L1 on BMDCs (Supplementary Fig. 6d). We also blocked the expression of PD-L1 on ex vivo-isolated CD11b^+^ SPDCs (Supplementary Fig. 6e). The kinetics of PD-1 expression on cocultured P14 T cells during proliferation were the same in every group, and blockade of PD-L1 on CL13-infected SPDCs could not restore the functionality of cocultured P14 T cells (Supplementary Fig. 6f–h).

These data indicated that LCMV-specific CD8^+^ T cells primed with DCs generated in CL13-infected mice could not efficiently produce effector cytokines and enzymes such as IFN-γ, IL-2 TNF-α and GzmB. In addition, this functional defect could not be alleviated by only blocking upregulated PD-L1 expressed on DCs. In summary, DCs differentiated during chronic CL13 infection play important roles in inducing both less functional cytotoxic CD8^+^ T cells and an increased number of Foxp3^+^ Treg cells.

Using two different models, we conclusively demonstrated that LCMV CL13-infected progenitor cells differentiate into less immunogenic DCs in vitro and in vivo and that these DCs induce less functional CD8^+^ T cells and more Foxp3^+^ Treg cells, resulting in a deficiency in immune responses.

### CD11b/c^+^ DCs generated during chronic viral infection could not induce functional effector CD8^+^ T cells in response to antigens other than involved in the chronic viral infection

**Fig. 5:**
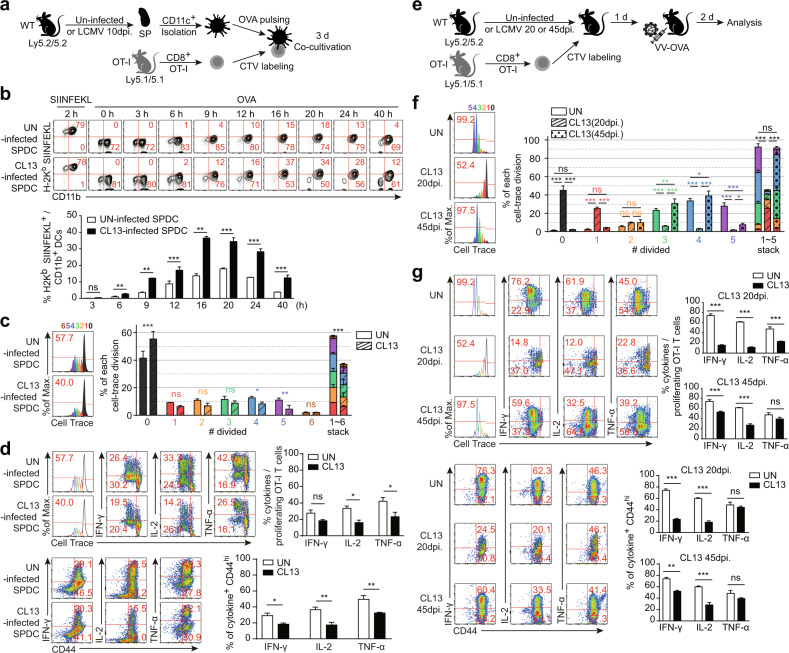
LCMV CL13-infected CD11b/c^+^ DCs cannot prime functional OT-I T cells specific for the antigens of additional infecting pathogens. **a** Experimental scheme of the in vitro OT-I T cell functional assay. SPDCs were isolated from naive and LCMV-infected mice. SPDCs were pulsed with the OVA protein (20 μg/ml) for 2 hours or OVA_257-264_ SIINFEKL peptide (0.2 μg/ml) for 2 hours and cocultured with CellTrace-labeled OT-I T cells. **b** Analysis of the capacity of each SPDC to take up, process and present exogenous OVA antigen by using an H-2K^b^-SIINFEKL complex-capturing antibody. The mean percentage of H-2K^b^-SIINFEKL-positive cells at each timepoint is shown in the bar graph. **c** The proliferation and division of OT-I T cells primed with OVA-pulsed SPDCs from uninfected or CL13-infected mice were analyzed by CellTrace dye staining. The bar graph presents the percentage of cell populations in each divided number. **d** Analysis of the effector function of cocultured OT-I T cells. For intracellular cytokine analysis, 0.5 μg/ml OVA_257-264_ SIINFEKL peptide was added to cultured cells for the last 5 hours. **e** Experimental scheme of the in vivo OT-I T cell functional assay. A total of 5 × 10^5^ OT-I cells were adoptively transferred by intravenous injection one day before VV-OVA infection. The mice in each group were infected with 2 × 10^6^ PFU of VV-OVA by intraperitoneal injection. **f** The proliferation and division of adoptively transferred OT-I T cells were analyzed by CellTrace dye staining. The bar graph presents the percentage of the cell populations in each division number. **g** The cytokine-secreting potential of adoptively transferred OT-I T cells was analyzed by intracellular cytokine staining and CellTrace dye staining. The kinetics of effector cytokine expression during OT-I T cell proliferation were analyzed (top three FACS plots and bar graphs). The expression levels of IFN-γ, IL-2, and TNF-α in activated CD44^hi^ proliferating OT-I T cells were investigated (bottom three FACS plots and bar graphs). The bar graphs show the means ± SDs (6 mice in each group). Each experiment was repeated twice. The *p* values in the figures indicate the following: *P < 0.05; **P < 0.01; **P < 0.01; ***P < 0.001.

As mentioned above, chronic exposure to viral antigens drives T cell dysfunction or exhaustion and ultimately suppresses the host immune system, resulting in failure to protect against infections by other types of pathogens. To investigate whether this T cell immune modulation is affected by DC function, we tested the proliferation and functional quality of CD8^+^ OT-I T cells in a chronic viral infection model. To this end, CellTrace-labeled CD8^+^ OT-I T cells were cocultured with OVA-pulsed CD11b/c^+^ SPDCs from either naive or LCMV CL13-infected mice (Fig. 5a). Before investigating the functionality of OT-I T cells, we tested the antigen uptake, processing, and presentation abilities of the DCs, which greatly influence antigen-specific T cell priming, by using an antibody against the OVA_257-264_ (SIINFEKL) peptide bound to H-2K^b^. After DCs were incubated with the SIINFEKL peptide in vitro for 2 hours, CD11c^+^-gated DCs were H-2K^b^-SIINFEKL peptide^+^, regardless of whether they were from naive mice or mice with acute or chronic infection. The level of the SIINFEKL peptide bound to H2-K^b^ was higher in LCMV-infected DCs incubated with the OVA protein than in naive DCs incubated with the OVA protein, and this increase persisted until after 20 hours of incubation. After more than 24 hours of incubation, the CD11b/c^+^ H-2K^b^-SIINFEKL^+^ cell proportion was decreased, and cell viability was reduced (Fig. 5b). These data indicated that chronic viral infection did not impair the abilities of DCs to take up, process, and present antigens. The expression levels of costimulatory and inhibitory molecules on the DC surface were almost identical to those shown in Fig. 3c–f. Next, we examined the phenotype and function of OT-I T cells cocultured with DCs. There were no marked differences in the expression of T cell activation markers, such as CD44 and CD69 (data not shown). Although the observed division number of proliferating OT-I cells was 6 for the UN and CL13-infected groups, the total proliferation of OT-I T cells primed with DCs from CL13-infected mice was significantly decreased (Fig. 5c). Moreover, the expression levels of effector cytokines such as IL-2 and TNF-α were reduced during the proliferation of OT-I T cells primed with DCs from CL13-infected mice, and the frequency of T cells expressing each cytokine in the activated CD44^hi^ population was reduced (Fig. 5d). In contrast to the results of the P14 T cell functional assay, the production of the three effector cytokines by OT-I T cells were not detectable by ELISA. These patterns of inhibition of T cell proliferation and cytokine expression were not observed upon coculture with ARM-infected SPDCs (Supplementary Fig. 7a, b). To further confirm these results in vivo, chronically infected mice were infected with an OVA-expressing vaccinia virus (VV-OVA), and CellTrace-labeled OT-I T cells were adoptively transferred into the mice. Then, two days after VV-OVA infection, OT-I T cells isolated from the spleen were analyzed (Fig. 5e). The proliferation and effector cytokine production of adoptively transferred OT-I T cells were extremely inhibited in mice coinfected with VV-OVA on Day 20 after CL13 infection compared to mice infected with only VV-OVA (Fig. 5f, g UN vs. CL13-20 dpi). We additionally performed functional analysis of OT-I T cells in mice coinfected with VV-OVA in the late phase of LCMV CL13 infection. Interestingly, the proliferation of OT-I T cells transferred into mice infected with VV-OVA on CL13 at 45 days post-infection recovered to that of the control group, but a functional defect in effector cytokine production was still observed at the late timepoint in the CL13 and VV-OVA coinfected group (Fig. 5f, g UN vs. CL13-45 dpi). Notably, OT-I T cells in the acute ARM-infected 20 dpi group also exhibited a slight defect in IFN-γ production, but the production of the other effector cytokines and the proliferation ratio of the cells were not affected. Furthermore, these defects in IFN-γ production were eliminated in the 45 dpi group (Supplementary Fig. 7c, d). These data suggested that the persistent viral load during chronic infection could modulate the action of myeloid DCs, ultimately resulting in alterations in T cell proliferation and function.

## Discussion

In this study, we induced chronic LCMV CL13 infection in B6 mice to test our experimental hypothesis. LCMV has been widely used for the study of the host immune response against acute or persistent viral infection because of its nonlytic characteristics, its genomic simplicity and the existence of two distinct strains for the establishment of acute and chronic viral infections. Although *Mus musculus* is the natural and primary host of LCMV, the LCMV infection model can serve as a model for studying human viruses because the immunologic network and events that occur during persistent viral infection are somewhat similar across species^55^. Observation of mice with LCMV infection for 30 days revealed that the differentiation of HSCs into progenitors was greatly restrained and that the development of CMPs and CDPs, which are directly related to myeloid DC differentiation, was suppressed in the BM of chronically CL13-infected mice. This restrained differentiation of the DC lineage was verified in a BMDC differentiation experiment. Despite the severely restrained differentiation of HSCs into progenitors in the BM during chronic viral infection, the composition and number of lineage-matured immune cells present in the BM were maintained to some degree. Hematopoiesis in viral infection is modulated by direct or indirect effects. Direct effects are induced by direct infection of HSCs or viral recognition. Indirect effects are induced by an altered BM environment with changes in inflammatory mediators and cells^56^. Since our data showed that the proportion of HSCs directly infected with CL13 was very low, the changes in the differentiation of HSCs into progenitor cells during CL13 infection were likely dependent on indirect effects. We did not further investigate key factors and conditions largely associated with restraining hematopoiesis; however, the regulation of hematopoiesis and DC development and subset alterations in DCs in a nonhomeostatic state, an emergency condition, have been studied recently^57–59^. Clonal expansion of certain HSCs or progenitors and alterations in the turnover rate of HSCs, the egress or migratory capacity of differentiated cells and the expression of chemokines in the BM should be investigated to clarify why hematopoiesis is altered in the chronically virus-infected state.

Although plasmacytoid DCs (pDCs) have been reported to play a crucial role in viral clearance by recognizing viral antigens through TLR7 or TLR9 and serving as the main source of type I IFNs (IFN-I)^60^, the major populations of LCMV-specific and LCMV-susceptible cells during chronic LCMV infection in our study were CD11b/c^+^ DCs, which showed weaker immunogenic characteristics than naive DCs and less sensitivity to further activation or maturation. We focused on MHC molecules, representative costimulatory and inhibitory molecules; however, the kinetics of the expression of other various molecules, such as TNF superfamily ligands, whose expression on APCs largely depends on type I and II interferons, need to be further analyzed because the expression of type I and II interferons is directly related to viral infection and T cell functionality^61^. Consequently, it would be helpful to understand the activation of DCs and the effects of DCs on T cell functionality during chronic viral infection. In addition to the expression of surface molecules, the expression of cytokines such as IL-6, IL-10, IL-12, TNF-α and TGF-β in CD11b^+^ DCs was altered during CL13 infection. The expression of chemokines such as CCL3 and CCL7 was also downregulated in CL13-infected CD11b^+^ DCs (data not shown). Decreases in the expression of these chemokines affect the recruitment or migration of T cells and other immune cells, resulting in further changes in immune responses^62,63^. In addition, CD11b^+^ myeloid DCs generated during chronic CL13 infection induced relatively normal proliferation of P14 T cells but led to defects in the effector cytokine production of these cells. Thus, cytokine production seemed to require more activating signals than proliferation. We further examined the expression of PD-l on P14 T cells and whether blockade of PD-L1 on CL13-infected CD11b^+^ DCs could restore cytokine production in P14 T cells since PD-L1 expression was upregulated in these DCs. Merely blocking upregulated PD-L1 expressed on CL13-infected DCs was insufficient to restore effector T cell functionality. Although we demonstrated that CD11b^+^ DCs from CL13-infected mice could not efficiently prime CD8^+^ T cells, the findings could not be directly applied to a systemic physiological model or explain the dysfunction of T cells in chronic viral infection because we used isolated naive antigen-specific CD8^+^ T cells, not exhausted CD8^+^ T cells induced during chronic viral infection in vivo. CD11b^+^ DCs produced during CL13 infection also had great potential to induce more Foxp3^+^ Treg cells. The expression of IDO, IL-10 and TGF-β in CL13-infected DCs was upregulated, and these molecules act as immunoregulatory factors that are crucial in the maintenance of Treg cells and long-term immune tolerance^64^.

During chronic viral infection, CD11b^+^ DCs repress T cell-mediated adaptive immune responses against newly encountered antigens as well as cognate viral antigens. Although CD11b^+^ DCs from CL13-infected mice took up, processed and presented antigens on their surface well, they did not efficiently prime OT-I T cells in vitro. Both the proliferation of OT-I T cells and the intracellular effector cytokine expression of these cells were significantly repressed when they were primed with CL13-infected myeloid DCs. Furthermore, we found that OT-I T cells adoptively transferred into VV-OVA-coinfected mice that were already chronically infected with CL13 had deficits in their proliferation and expression of effector cytokines. Interestingly, only the proliferation of OT-I T cells was rescued in the late phase (CL13-45 dpi) of chronic infection. This difference in the severity of T cell dysfunction likely resulted from the different viral loads at different timepoints because the immune response and tissue distribution of T cells may be affected by the viral load^65–68^. The viral titers of CL13 in various murine organs and tissues decline over time^25^; in particular, the viral titer in splenic CD11b^+^ DCs at 30 days post-CL13 infection was half of that at 15 days post-CL13 infection (data not shown). Unfortunately, this VV-OVA infection model has a limitation: adoptively transferred OT-I T cells not only interact with CD11b^+^ DCs but are also affected by other systemic immune responses in vivo.

In our study, we focused on the qualitative differences between CD11b/c^+^ DCs differentiated during chronic infection or steady-state conditions. Conventional CD11c^+^ DCs (cDCs) can be divided into the cDC1 subset and the cDC2 subset, which comprises CD11b^+^ DCs. In general, the priming of CD8^+^ T cells is mediated mainly by the cDC1 subset, and a previous study investigated how a cDC1 population exposed to a chronic LCMV antigen or directly infected with a chronic LCMV strain affects T cell immune responses, focusing on induced IL-10 expression in CD4^+^ T cells^69^. In contrast, in our study, the cDC2 subset also effectively influenced the functional alterations in CD8^+^ T cells during persistent viral infection. Thus, all types of DCs and DC progenitors are affected by infection, and each plays a specific role against chronic infection, thereby modulating the immune threshold and T cell function. In accordance with this idea, the role of cDCs and their interaction with pDCs during viral infections, such as those caused by vaccinia virus, have been previously studied^70^. By characterizing CD11b^+^ DCs and performing functional analysis of effector T cells specific for cognate virus or newly encountered antigens, we demonstrated that CD11b^+^ DCs generated during chronic viral infection were less immunogenic and could contribute to adaptive immune dysfunction. In this study, we did not target a single factor or reveal a specific mechanism; however, various factors we assessed in CD11b^+^ DCs were found to work in combination and cause immune suppression during chronic viral infection. The finding that simply blocking PD-L1 did not restore T cell dysfunction may provide evidence for this combined effect.

While T cells are functionally repressed by CD11b^+^ DCs during chronic infection, the NK cell-mediated immune response can be enhanced during CL13 infection, and activation of the innate immune response during chronic infection can even delay tumor progression^71,72^. This enhancement of innate immunity in the presence of a persistent antigen can be explained by the concept of innate memory, that is, ‘trained immunity’, another unique host defense mechanism^73^. Similarly, some studies have reported that coinfection with pathogen expressing different antigens during persistent or latent infection may have a beneficial effect on the host immune response^74–76^. Thus, DCs and T cells, in addition to other immune cell populations such as innate immune cells and lymphoid stromal cells, may be influenced by infection, and all these cells may create complex immune networks that induce distinct, not-yet-elucidated immune responses during persistent infection. Although we revealed the general potential of CD11b^+^ DCs to contribute to immune suppression during chronic viral infection, our study demonstrated a single simple but clear mechanism involving modulation of DCs and DC progenitors that may underlie the immune dysfunction and suppression accompanied by functional alterations in T cells during chronic viral infection. Furthermore, our study also demonstrated that the less immunogenic DCs generated from the BM during chronic viral infection may contribute to insufficient T cell immune responses against newly invading pathogens by limiting optimized T cell priming and differentiation.

## Supplementary information

Supplementary Figures

## References

[1] Rehermann B, Nascimbeni M (2005). Immunology of hepatitis B virus and hepatitis C virus infection. Nat. Rev. Immunol..

[2] Virgin HW, Wherry EJ, Ahmed R (2009). Redefining chronic viral infection. Cell.

[3] Blank CU (2019). Defining ‘T cell exhaustion’. Nat. Rev. Immunol..

[4] Daniel B (2022). Divergent clonal differentiation trajectories of T cell exhaustion. Nat. Immunol..

[5] Im SJ, Ha SJ (2020). Re-defining T-cell exhaustion: subset, function, and regulation. Immune Netw..

[6] Jin HT, Jeong YH, Park HJ, Ha SJ (2011). Mechanism of T cell exhaustion in a chronic environment. BMB Rep..

[7] Barber DL (2006). Restoring function in exhausted CD8 T cells during chronic viral infection. Nature.

[8] Schietinger A, Greenberg PD (2014). Tolerance and exhaustion: defining mechanisms of T cell dysfunction. Trends Immunol..

[9] Xu YD, Cheng M, Shang PP, Yang YQ (2022). Role of IL-6 in dendritic cell functions. J. Leukoc. Biol..

[10] Steinman RM (2012). Decisions about dendritic cells: past, present, and future. Annu. Rev. Immunol..

[11] He X, Xu C (2020). Immune checkpoint signaling and cancer immunotherapy. Cell Res.

[12] Mbongue JC (2015). The role of indoleamine 2, 3-dioxygenase in immune suppression and autoimmunity. Vaccines (Basel).

[13] Taylor A, Verhagen J, Blaser K, Akdis M, Akdis CA (2006). Mechanisms of immune suppression by interleukin-10 and transforming growth factor-beta: the role of T regulatory cells. Immunology.

[14] Yoo S, Ha SJ (2016). Generation of tolerogenic dendritic cells and their therapeutic applications. Immune Netw..

[15] McNamara LA, Collins KL (2011). Hematopoietic stem/precursor cells as HIV reservoirs. Curr. Opin. HIV AIDS.

[16] Matatall KA (2016). Chronic infection depletes hematopoietic stem cells through stress-induced terminal differentiation. Cell Rep..

[17] Glatman Zaretsky A, Engiles JB, Hunter CA (2014). Infection-induced changes in hematopoiesis. J. Immunol..

[18] Carter CC (2010). HIV-1 infects multipotent progenitor cells causing cell death and establishing latent cellular reservoirs. Nat. Med..

[19] Said EA (2010). Programmed death-1-induced interleukin-10 production by monocytes impairs CD4+ T cell activation during HIV infection. Nat. Med..

[20] Ha SJ (2008). Enhancing therapeutic vaccination by blocking PD-1-mediated inhibitory signals during chronic infection. J. Exp. Med.

[21] Randall TD, Weissman IL (1998). Characterization of a population of cells in the bone marrow that phenotypically mimics hematopoietic stem cells: resting stem cells or mystery population?. Stem Cells.

[22] Frascoli, M., Proietti, M. & Grassi, F. Phenotypic analysis and isolation of murine hematopoietic stem cells and lineage-committed progenitors. *J. Vis. Exp*. 10.3791/3736 (2012). 10.3791/3736 PMC3471276 22805770

[23] Onai N, Manz MG, Schmid MA (2010). Isolation of common dendritic cell progenitors (CDP) from mouse bone marrow. Methods Mol. Biol..

[24] Alexaki A, Liu Y, Wigdahl B (2008). Cellular reservoirs of HIV-1 and their role in viral persistence. Curr. HIV Res..

[25] Blackburn SD (2010). Tissue-specific differences in PD-1 and PD-L1 expression during chronic viral infection: implications for CD8 T-cell exhaustion. J. Virol..

[26] Fogg DK (2006). A clonogenic bone marrow progenitor specific for macrophages and dendritic cells. Science.

[27] Inaba K (1992). Generation of large numbers of dendritic cells from mouse bone marrow cultures supplemented with granulocyte/macrophage colony-stimulating factor. J. Exp. Med..

[28] Lutz MB (1999). An advanced culture method for generating large quantities of highly pure dendritic cells from mouse bone marrow. J. Immunol. Methods.

[29] Mellor AL (2003). Cutting edge: induced indoleamine 2,3 dioxygenase expression in dendritic cell subsets suppresses T cell clonal expansion. J. Immunol..

[30] Mellor AL, Lemos H, Huang L (2017). Indoleamine 2,3-Dioxygenase and Tolerance: Where Are We Now?. Front. Immunol..

[31] Mellor AL, Munn DH (2004). IDO expression by dendritic cells: tolerance and tryptophan catabolism. Nat. Rev. Immunol..

[32] Xie FT (2015). IDO expressing dendritic cells suppress allograft rejection of small bowel transplantation in mice by expansion of Foxp3+ regulatory T cells. Transpl. Immunol..

[33] Harden JL, Egilmez NK (2012). Indoleamine 2,3-dioxygenase and dendritic cell tolerogenicity. Immunol. Investig..

[34] Sanjabi S, Zenewicz LA, Kamanaka M, Flavell RA (2009). Anti-inflammatory and pro-inflammatory roles of TGF-beta, IL-10, and IL-22 in immunity and autoimmunity. Curr. Opin. Pharmacol..

[35] Chen Y, Kuchroo VK, Inobe J, Hafler DA, Weiner HL (1994). Regulatory T cell clones induced by oral tolerance: suppression of autoimmune encephalomyelitis. Science.

[36] Montagnoli C (2002). B7/CD28-dependent CD4+CD25+ regulatory T cells are essential components of the memory-protective immunity to Candida albicans. J. Immunol..

[37] Moore KW, de Waal Malefyt R, Coffman RL, O’Garra A (2001). Interleukin-10 and the interleukin-10 receptor. Annu. Rev. Immunol..

[38] Morelli AE, Thomson AW (2007). Tolerogenic dendritic cells and the quest for transplant tolerance. Nat. Rev. Immunol..

[39] Couper KN, Blount DG, Riley EM (2008). IL-10: the master regulator of immunity to infection. J. Immunol..

[40] Brooks DG (2006). Interleukin-10 determines viral clearance or persistence in vivo. Nat. Med..

[41] Pulendran B (2005). Variegation of the immune response with dendritic cells and pathogen recognition receptors. J. Immunol..

[42] Krementsov DN, Thornton TM, Teuscher C, Rincon M (2013). The emerging role of p38 mitogen-activated protein kinase in multiple sclerosis and its models. Mol. Cell. Biol..

[43] Kim HS (2016). Curdlan activates dendritic cells through dectin-1 and toll-like receptor 4 signaling. Int. Immunopharmacol..

[44] Saraiva M, O’Garra A (2010). The regulation of IL-10 production by immune cells. Nat. Rev. Immunol..

[45] Chi H (2006). Dynamic regulation of pro- and anti-inflammatory cytokines by MAPK phosphatase 1 (MKP-1) in innate immune responses. Proc. Natl Acad. Sci. USA..

[46] Wang W, Li J, Wu K, Azhati B, Rexiati M (2016). Culture and identification of mouse bone marrow-derived dendritic cells and their capability to induce T lymphocyte proliferation. Med. Sci. Monit..

[47] Vijh S, Pilip IM, Pamer EG (1998). Effect of antigen-processing efficiency on in vivo T cell response magnitudes. J. Immunol..

[48] Mach N (2000). Differences in dendritic cells stimulated in vivo by tumors engineered to secrete granulocyte-macrophage colony-stimulating factor or Flt3-ligand. Cancer Res..

[49] Pardoll DM (2012). The blockade of immune checkpoints in cancer immunotherapy. Nat. Rev. Cancer.

[50] Wykes MN, Lewin SR (2018). Immune checkpoint blockade in infectious diseases. Nat. Rev. Immunol..

[51] McClanahan F (2015). PD-L1 checkpoint blockade prevents immune dysfunction and leukemia development in a mouse model of chronic lymphocytic leukemia. Blood.

[52] Hassannia H (2020). Blockage of immune checkpoint molecules increases T-cell priming potential of dendritic cell vaccine. Immunology.

[53] Dammeijer F (2020). The PD-1/PD-L1-checkpoint restrains T cell immunity in tumor-draining lymph nodes. Cancer Cell.

[54] Peng Q (2020). PD-L1 on dendritic cells attenuates T cell activation and regulates response to immune checkpoint blockade. Nat. Commun..

[55] Ng CT, Snell LM, Brooks DG, Oldstone MB (2013). Networking at the level of host immunity: immune cell interactions during persistent viral infections. Cell Host Microbe.

[56] Pascutti MF, Erkelens MN, Nolte MA (2016). Impact of viral infections on hematopoiesis: from beneficial to detrimental effects on bone marrow output. Front. Immunol..

[57] Lin DS (2021). Single-cell analyses reveal the clonal and molecular aetiology of Flt3L-induced emergency dendritic cell development. Nat. Cell Biol..

[58] Bosteels C (2020). Inflammatory Type 2 cDCs Acquire Features of cDC1s and Macrophages to Orchestrate Immunity to Respiratory Virus Infection. Immunity.

[59] Isringhausen S (2021). Chronic viral infections persistently alter marrow stroma and impair hematopoietic stem cell fitness. J. Exp. Med.

[60] Swiecki M, Gilfillan S, Vermi W, Wang Y, Colonna M (2010). Plasmacytoid dendritic cell ablation impacts early interferon responses and antiviral NK and CD8(+) T cell accrual. Immunity.

[61] Wang KC, Chu KL, Batista NV, Watts TH (2018). Conserved and differential features of TNF Superfamily ligand expression on APC subsets over the course of a chronic viral infection in mice. Immunohorizons.

[62] Oelkrug C, Ramage JM (2014). Enhancement of T cell recruitment and infiltration into tumours. Clin. Exp. Immunol..

[63] Oo YH, Shetty S, Adams DH (2010). The role of chemokines in the recruitment of lymphocytes to the liver. Dig. Dis..

[64] Levings MK, Bacchetta R, Schulz U, Roncarolo MG (2002). The role of IL-10 and TGF-beta in the differentiation and effector function of T regulatory cells. Int. Arch. Allergy Immunol..

[65] Wherry EJ, Blattman JN, Murali-Krishna K, van der Most R, Ahmed R (2003). Viral persistence alters CD8 T-cell immunodominance and tissue distribution and results in distinct stages of functional impairment. J. Virol..

[66] Fuller MJ, Zajac AJ (2003). Ablation of CD8 and CD4 T cell responses by high viral loads. J. Immunol..

[67] Wherry EJ, Ahmed R (2004). Memory CD8 T-cell differentiation during viral infection. J. Virol..

[68] Wherry EJ, Blattman JN, Ahmed R (2005). Low CD8 T-cell proliferative potential and high viral load limit the effectiveness of therapeutic vaccination. J. Virol..

[69] Baca Jones C (2014). Direct infection of dendritic cells during chronic viral infection suppresses antiviral T cell proliferation and induces IL-10 expression in CD4 T cells. PLoS One.

[70] Brewitz A (2017). CD8(+) T cells orchestrate pDC-XCR1(+) dendritic cell spatial and functional cooperativity to optimize priming. Immunity.

[71] Oh JH (2019). Sustained type I interferon reinforces nk cell-mediated cancer immunosurveillance during chronic virus infection. Cancer Immunol. Res.

[72] Bukowski JF, Biron CA, Welsh RM (1983). Elevated natural killer cell-mediated cytotoxicity, plasma interferon, and tumor cell rejection in mice persistently infected with lymphocytic choriomeningitis virus. J. Immunol..

[73] Netea MG (2020). Defining trained immunity and its role in health and disease. Nat. Rev. Immunol..

[74] Tillmann HL (2001). Infection with GB virus C and reduced mortality among HIV-infected patients. N. Engl. J. Med..

[75] Xiang J (2001). Effect of coinfection with GB virus C on survival among patients with HIV infection. N. Engl. J. Med..

[76] Barton ES (2007). Herpesvirus latency confers symbiotic protection from bacterial infection. Nature.

