# Supplementary material for: Chronic LCMV infection regulates the effector T cell response by inducing the generation of less immunogenic dendritic cells
Source: Exp Mol Med. 2023 May 1;55(5):999–1012. doi: 10.1038/s12276-023-00991-5 (PMC10238507; doi:10.1038/s12276-023-00991-5)
Supplement: Supplementary file 1 — Supplementary Figures [file 12276_2023_991_MOESM1_ESM.pdf]

Supplementary Fig. 1

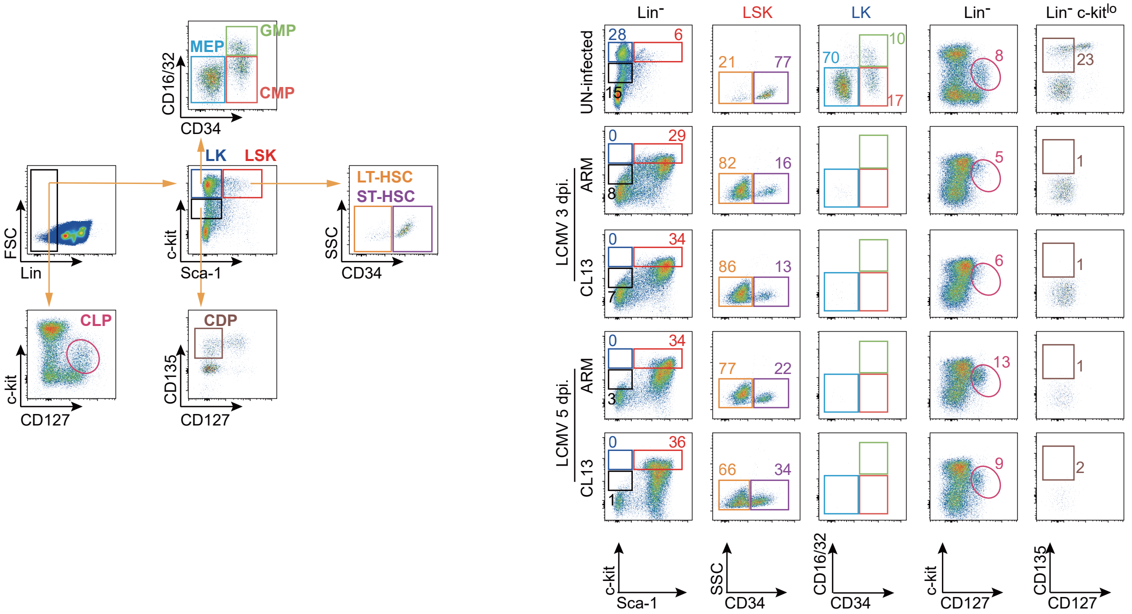

Supplementary Fig. 1. Analysis of HSC and progenitor populations in the BM on Days 3 and 5 after LCMV infection.

HSCs and progenitor cells were identified based on surface molecule expression, as described in the gating strategy for the lineage marker-negative population. On Days 3 and 5 after LCMV infection, BM cells were isolated, and the percentage of each HSC and progenitor population was analyzed (5 mice in each group).

Supplementary Fig. 2

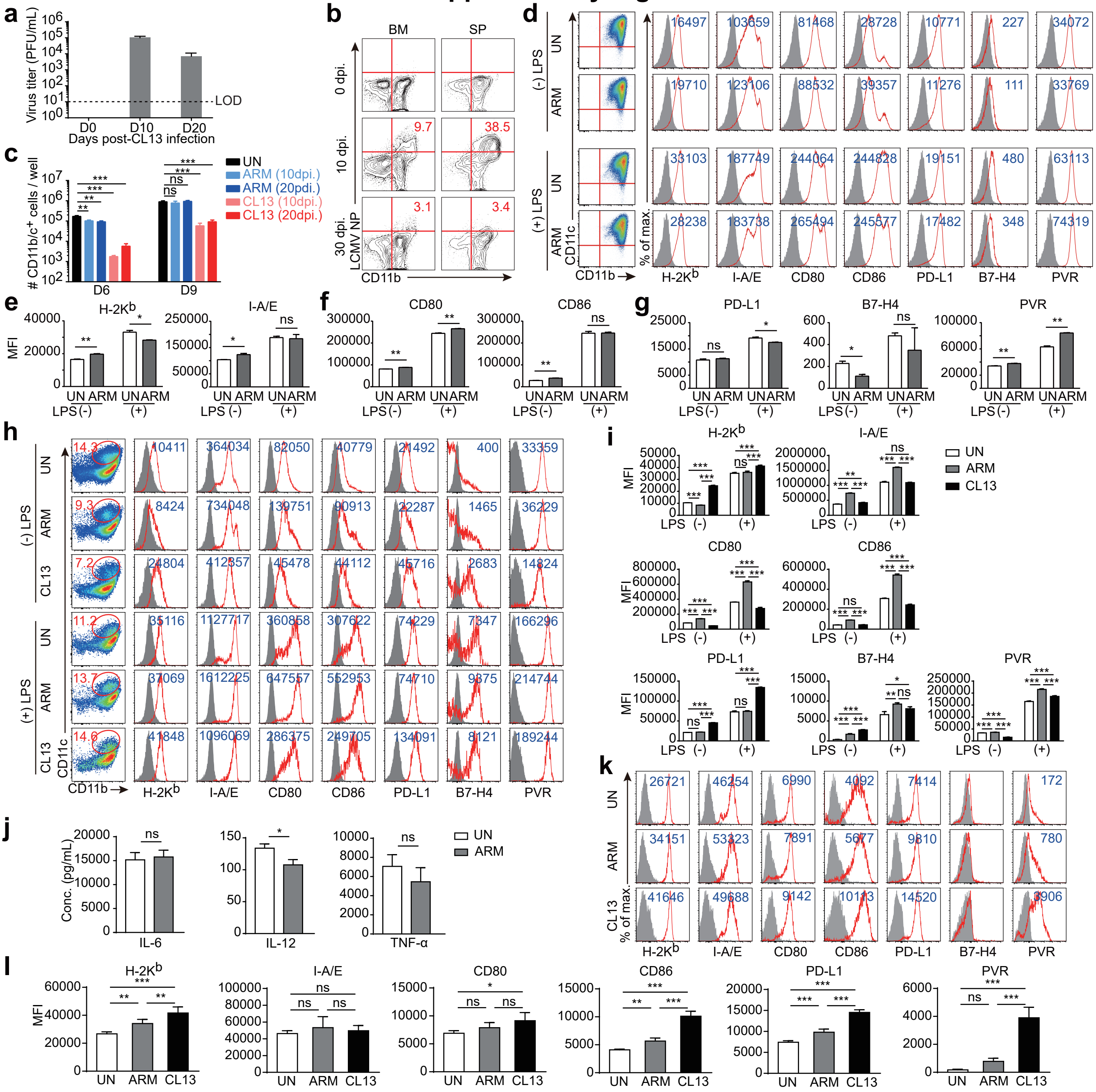

Supplementary Fig. 2. Phenotypic analysis of BMDCs differentiated from ARM-infected mice and *ex vivo* splenic CD11b$^+$ DCs. [a] The LCMV CL13 viral titer in the BM was analyzed by a plaque formation assay. **[b]** The frequency of the LCMV NP$^+$ CD11b$^+$ population in the BM was analyzed at 0, 10 and 30 days after LCMV CL13 infection. **[c]** Number of CD11b/c$^+$ cells on Days 6 and 9 of BMDC differentiation. **[d]** Flow cytometry analysis of surface molecules on UN-BMDCs and ARM-infected BMDCs on Day 10 of BMDC differentiation. The histograms in the figure show the MFI. **[e-g]** Bar plot showing the MFI values of **[e]** MHCs, **[f]** costimulatory and **[g]** inhibitory molecules. **[h]** Expression of surface molecules on CD11b/c$^+$ cells on Day 4 of BMDC differentiation. **[i]** The bar graph presents the MFI values of each molecule on CD11b/c$^+$ cells on Day 4 of BMDC differentiation. **[j]** The concentrations of inflammatory cytokines secreted by UN-BMDCs or ARM-infected BMDCs were measured by ELISA. **[k]** The histograms present the expression of each surface molecule on splenic CD11b$^+$ DCs after 10 days of LCMV infection. **[l]** The bar plots present the MFI values of each surface molecule on CD11b$^+$ splenic DCs. The bar graphs show the means ± SDs (n = 3 samples). Each experiment was repeated 3 times. The *p* values in the figures indicate the following: *P < 0.05; **P < 0.01; **P < 0.01; ***P < 0.001

Supplementary Fig. 3

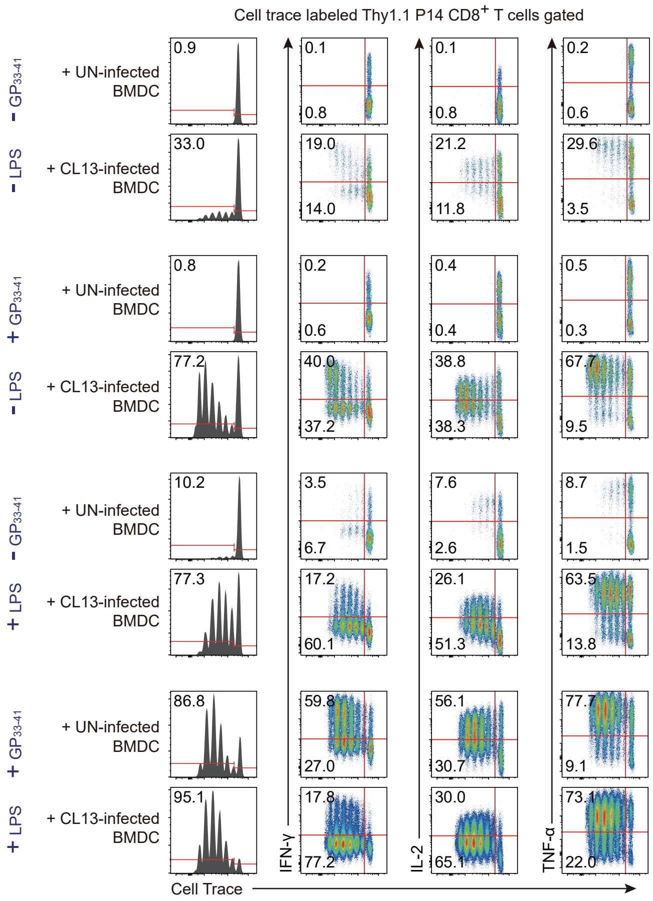

Supplementary Fig. 3. Analysis of the proliferation and effector functions of LCMV-specific CD8$^+$ P14 T cells primed with BMDCs stimulated under various conditions. CD8$^+$ T cells isolated from the spleen of naïve P14 TCR-transgenic mice were cocultured with BMDCs generated from naïve or CL13-infected mice for 3 days. The cells were treated with (+) or without (-) LPS and $GP_{33\text{-}41}$ peptides.

Supplementary Fig. 4

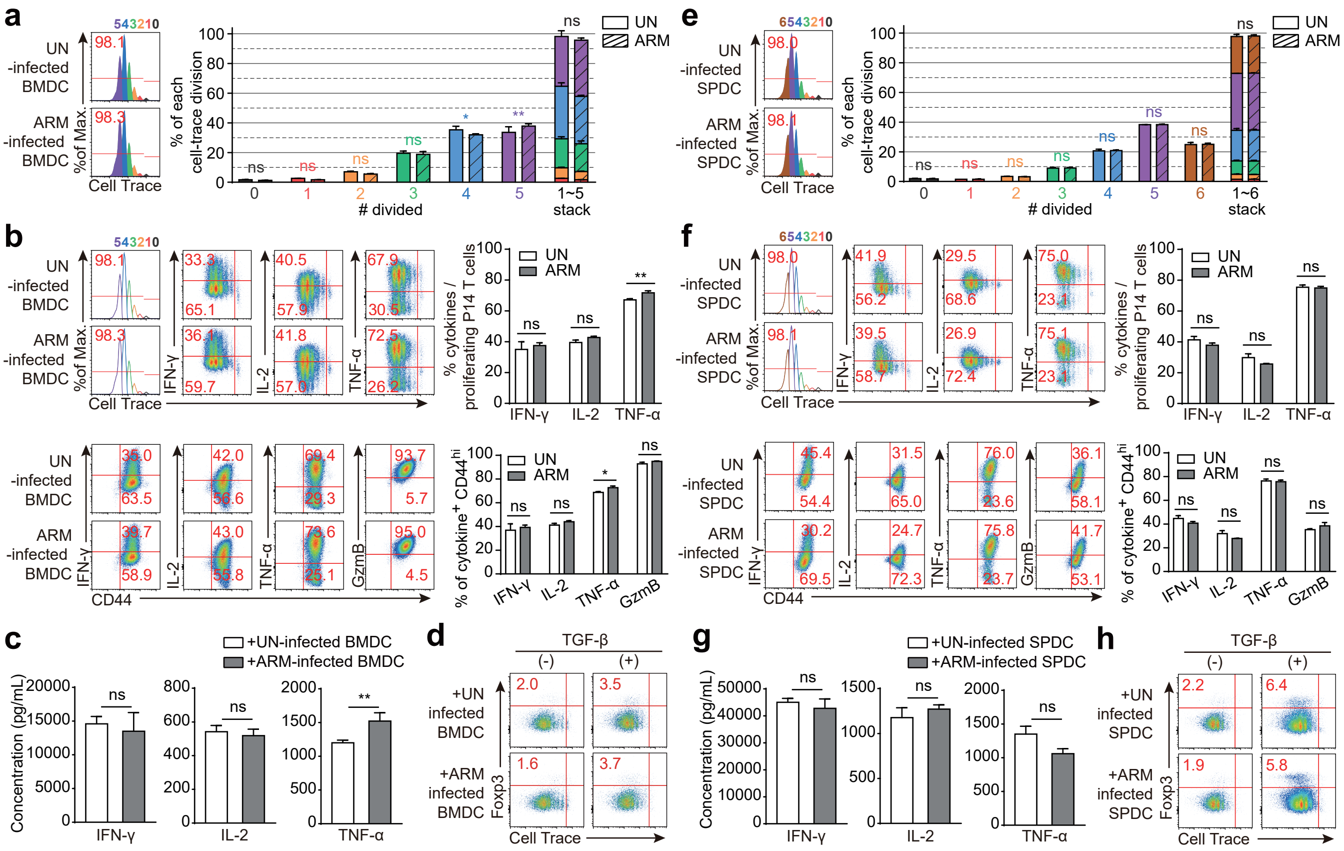

Supplementary Fig. 4. Analysis of P14 CD8^{+} T cell functionality and induced Foxp3^{+} Treg cell population primed with ARM-infected DCs. **[a]** The proliferation and division of P14 T cells cocultured with UN-BMDCs or ARM-infected BMDCs were analyzed by CellTrace dye staining. The bar graph presents the percentage of the cell populations in each division number. **[b]** The potential cytokine secretion of P14 T cells was analyzed by intracellular cytokine staining and CellTrace dye staining. The kinetics of effector cytokine expression during P14 T cell proliferation were analyzed (top two FACS plots and bar graph). The expression levels of IFN-γ, IL-2, TNF-α and granzyme B in activated CD44^{hi} proliferating P14 T cells were investigated (bottom two FACS plots and bar graph). **[c]** The total amount of each cytokine expressed in the supernatant of P14-BMDC cocultures was measured by a multiplex bead assay. **[d]** Conversion ratio of Foxp3^{+} Treg cells in a coculture containing CD4^{+} T cells with UN-BMDCs or ARM-infected BMDCs in each condition. **[e]** The proliferation and division of P14 T cells cocultured with *ex vivo* SPDCs from uninfected or ARM-infected mice were analyzed. The bar graph presents the percentages of the cell populations in each division number. **[f]** The cytokine-secreting potential of P14 T cells was analyzed. The kinetics of effector cytokine expression during P14 T cell proliferation were analyzed (top two FACS plots and bar graph). The expression levels of IFN-γ, IL-2, TNF-α and GzmB in activated CD44^{hi} proliferating P14 T cells were investigated (bottom two FACS plots and bar graph). **[g]** The total amount of each cytokine expressed in the supernatant of P14-SPDC cocultures was measured by a multiplex bead assay. **[h]** Conversion ratio of Foxp3^{+} Treg cells derived from CD4^{+} T cells cocultured with SPDCs from uninfected or ARM-infected mice in each condition. The bar graphs show the means ± SDs (6 mice in each group). Each experiment was repeated twice. The *p* values in the figures indicate the following: $*P < 0.05$; $**P < 0.01$; $**P < 0.01$; $***P < 0.001$

Supplementary Fig. 5

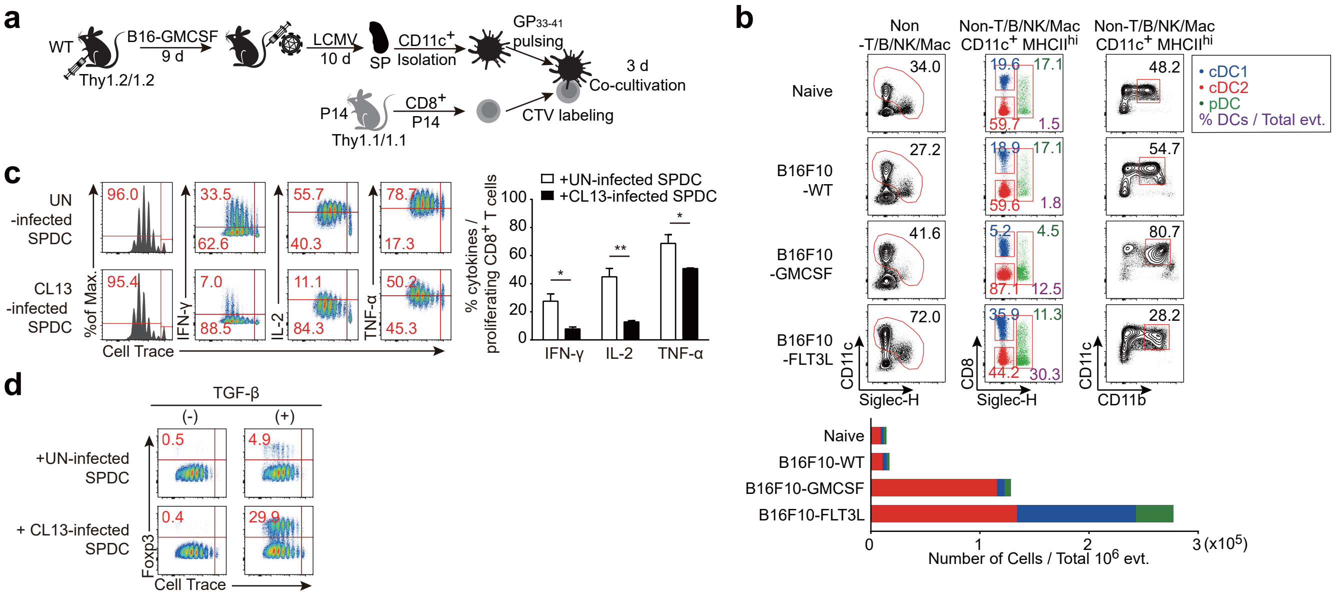

Supplementary Fig. 5. Analysis of P14 CD8^{+} T cell functionality after priming with CD11b^{+} SPDCs selectively expanded by GM-CSF-expressing tumor cells during CL13 chronic infection. [a] Experimental scheme for manipulating CD11b^{+} DCs in the spleen with GM-CSF expressing B16F10 cells. **[b]** Expansion of each subset of SPDCs with B16F10 cells engineered to express GM-CSF or Flt3L. The percentages (top, flow cytometry plot) and numbers (bottom, bar plot) of cDC1s, cDC2s and pDCs in the spleen on Day 9 after injection of each tumor cell line. The injection of B16F10-GM-CSF cells selectively expanded the CD11b^{+} cDC2 population. **[c]** Proliferation and effector cytokine expression analysis of P14 cells primed with CD11b^{+} SPDCs expanded by injection with GM-CSF-expressing B16F10 cells. **[d]** Analysis of induced Foxp3^{+} Treg cells derived from CD4^{+} T cells cocultured with CD11b^{+} SPDCs expanded by injection of B16F10-GMCSF cells. The bar graphs show the means ± SDs (3 mice in each group). Each experiment was repeated twice. The p values in the figures indicate the following: *P < 0.05; **P < 0.01; **P < 0.01; ***P < 0.001

Supplementary Fig. 6.

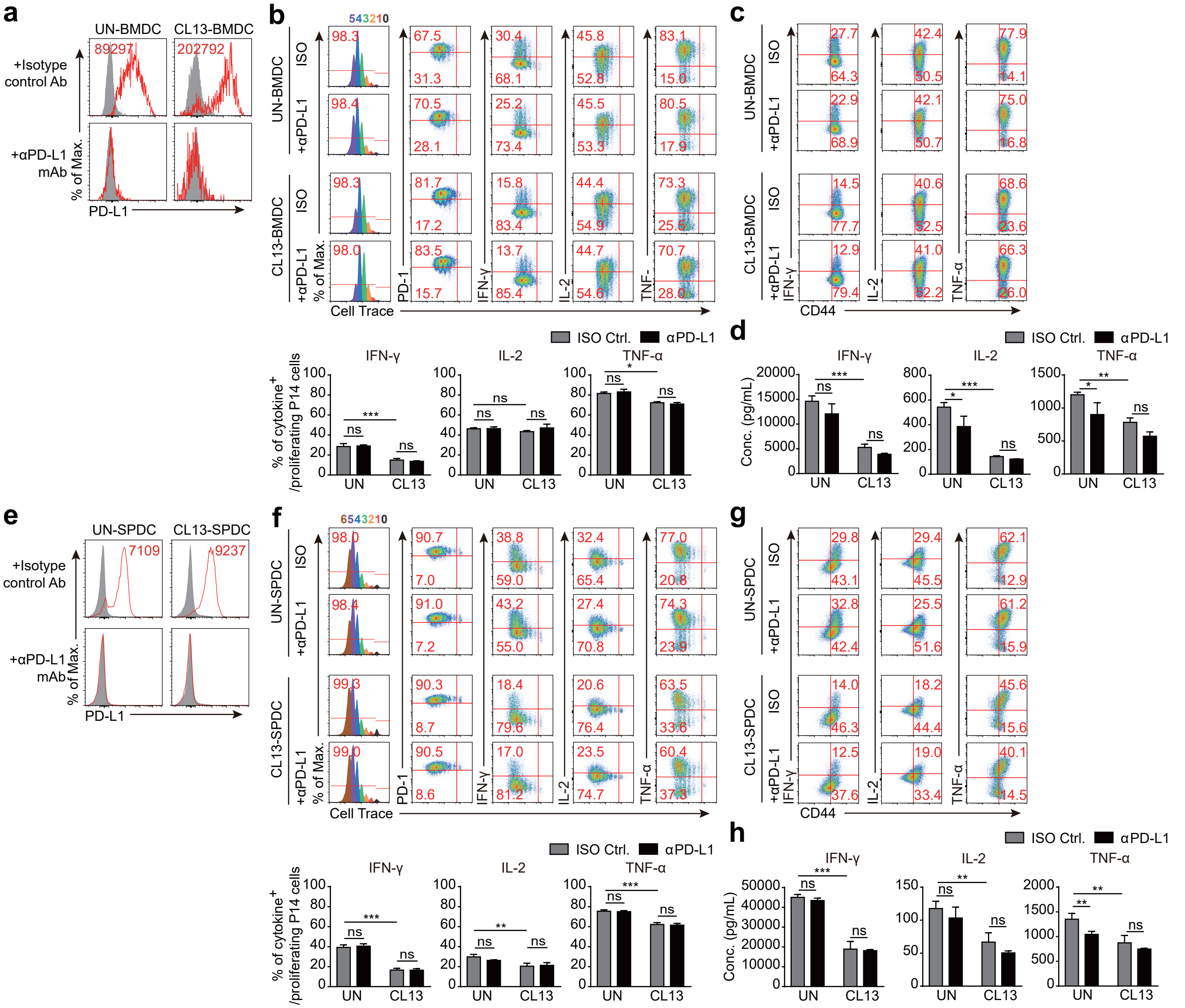

Supplementary Fig. 6. Functional analysis of P14 T cells primed with CD11b⁺ myeloid DCs in the context of PD-L1 blockade. [a] The blockade of PD-L1 on the surface of BMDCs after treatment with a purified anti-PD-L1 antibody was verified by flow cytometry analysis. **[b]** The kinetics of the expression of various molecules, such as PD-1, IFN-γ, IL-2 and TNF-α, in P14 T cells cocultured with BMDCs were investigated. **[c]** Upon coculture with BMDCs, the frequency of each effector cytokine-expressing $CD44^{hi}$ activated P14 T cell population was analyzed in each condition. **[d]** The total amount of each effector cytokine in the supernatant of P14-BMDC cocultures was estimated by a multiplex bead assay. **[e]** The blockade of PD-L1 on the surface of SPDCs after treatment with purified anti-PD-L1 antibody was verified by flow cytometry analysis. **[f]** The kinetics of the expression of various molecules, such as PD-1, IFN-γ, IL-2 and TNF-α, in P14 T cells cocultured with SPDCs were assessed. **[g]** Upon coculture with SPDCs, the frequency of each effector cytokine-expressing $CD44^{hi}$ activated P14 T cell population was analyzed in each condition. **[h]** The total amount of each effector cytokine in the supernatant of P14-SPDC cocultures was estimated by a multiplex bead assay. The bar graphs show the means ± SDs (3 mice in each group). Each experiment was repeated twice. The *p* values in the figures indicate the following: $*P < 0.05$; $**P < 0.01$; $**P < 0.01$; $***P < 0.001$

Supplementary Fig. 7

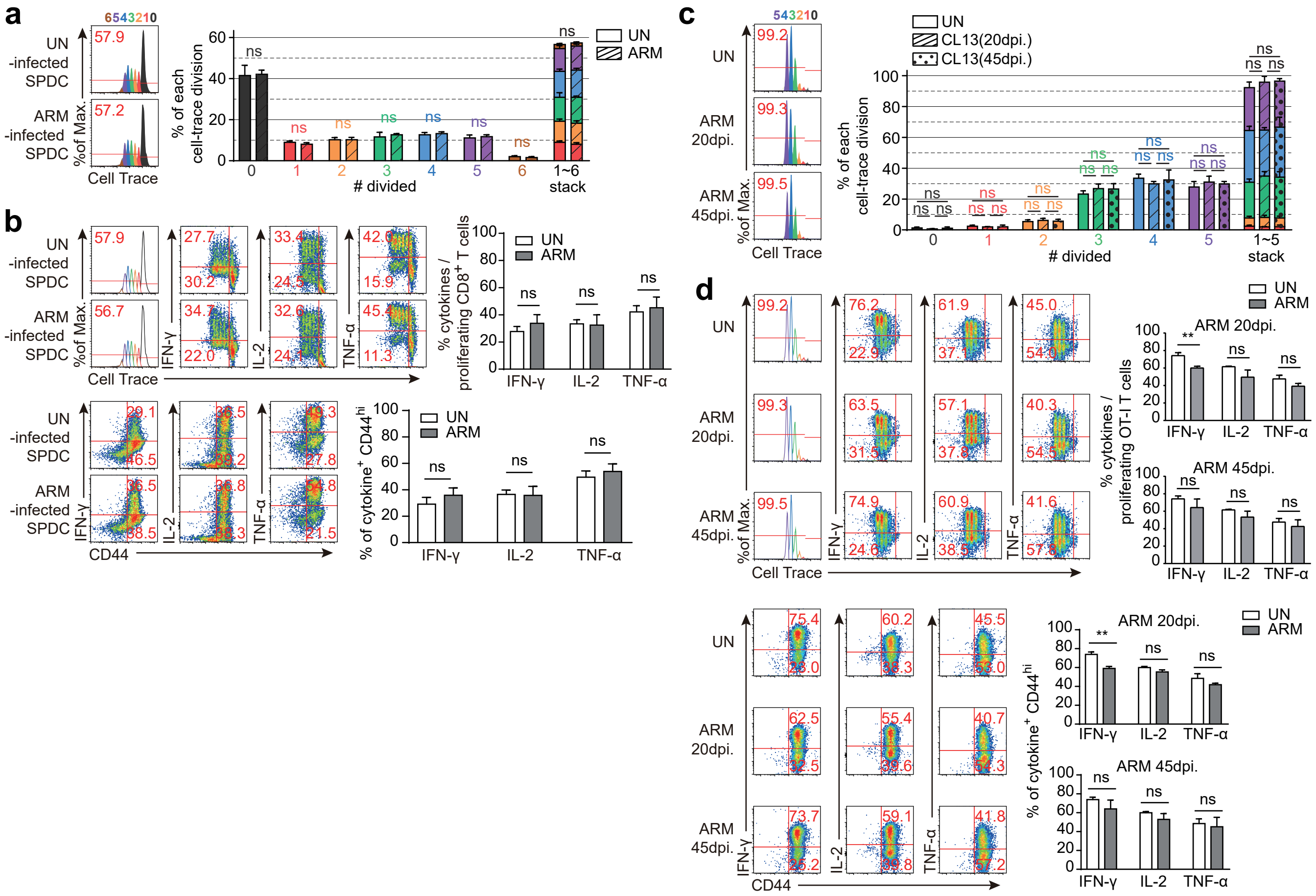

Supplementary Fig. 7. Functional analysis of OT-I CD8^{+} T cells primed with ARM-infected DCs. [a] The *in vitro* proliferation and division of OT-I T cells cocultured with OVA-pulsed naïve or ARM-infected SPDCs were analyzed by CellTrace dye staining. The bar graph presents the percentages of the cell populations in each division number. **[b]** The potential cytokine secretion of OT-I T cells cocultured with OVA-pulsed naïve or ARM-infected SPDCs was analyzed by intracellular cytokine staining and CellTrace dye staining. The kinetics of effector cytokine expression during OT-I T cell proliferation were analyzed (top two FACS plots and bar graph). The expression levels of IFN-γ, IL-2, and TNF-α in activated CD44^{hi} proliferating OT-I T cells were investigated (bottom two FACS plots and bar graph). **[c]** Analysis of the proliferation and division of adoptively transferred OT-I T cells in the spleen of uninfected or ARM-infected mice after VV-OVA coinfection. The bar graph presents the percentages of the cell populations in each division number. **[d]** The cytokine-secreting potential of adoptively transferred OT-I T cells was analyzed by intracellular cytokine staining and CellTrace dye staining. The kinetics of effector cytokine expression during OT-I T cell proliferation were analyzed (top three FACS plots and bar graphs). The expression levels of IFN-γ, IL-2, and TNF-α in activated CD44^{hi} proliferating OT-I T cells were investigated (bottom three FACS plots and bar graphs). The bar graphs show the means ± SDs (6 mice in each group). Each experiment was repeated twice. The *p* values in the figures indicate the following: *P < 0.05; **P < 0.01; **P < 0.01; ***P < 0.001

Supplementary Fig. 1. Analysis of HSC and progenitor populations in the BM on Days 3 and 5 after LCMV infection.

HSCs and progenitor cells were identified based on surface molecule expression, as described in the gating strategy for the lineage marker-negative population. On Days 3 and 5 after LCMV infection, BM cells were isolated, and the percentage of each HSC and progenitor population was analyzed (5 mice in each group).

Supplementary Fig. 2. Phenotypic analysis of BMDCs differentiated from ARM-infected mice and *ex vivo* splenic CD11b$^+$ DCs.

[a] The LCMV CL13 viral titer in the BM was analyzed by a plaque formation assay.

[**b]** The frequency of the LCMV NP$^+$ CD11b$^+$ population in the BM was analyzed at 0, 10 and 30 days after LCMV CL13 infection.

[**c]** Number of CD11b/c$^+$ cells on Days 6 and 9 of BMDC differentiation.

[**d]** Flow cytometry analysis of surface molecules on UN-BMDCs and ARM-infected BMDCs on Day 10 of BMDC differentiation. The histograms in the figure show the MFI.

[e-g] Bar plot showing the MFI values of [**e]** MHC, [**f]** costimulatory and [**g]** inhibitory molecules.

[**h]** Expression of surface molecules on CD11b/c$^+$ cells on Day 4 of BMDC differentiation.

[**i]** The bar graph presents the MFI values of each molecule on CD11b/c$^+$ cells on Day 4 of BMDC differentiation.

[j] The concentrations of inflammatory cytokines secreted by UN-BMDCs or ARM-infected BMDCs were measured by ELISA.

[k] The histograms present the expression of each surface molecule on splenic $CD11b^+$ DCs after 10 days of LCMV infection.

[l] The bar plots present the MFI values of each surface molecule on $CD11b^+$ splenic DCs.

The bar graphs show the means ± SDs (n = 3 samples). Each experiment was repeated 3 times. The *p* values in the figures indicate the following: *P < 0.05; **P < 0.01; **P < 0.01; ***P < 0.001.

Supplementary Fig. 3. Analysis of the proliferation and effector functions of LCMV-specific $CD8^+$ P14 T cells primed with BMDCs stimulated under various conditions.

$CD8^+$ T cells isolated from the spleen of naïve P14 TCR-transgenic mice were cocultured with BMDCs generated from naïve or CL13-infected mice for 3 days. The cells were treated with (+) or without (-) LPS and $GP_{33\text{-}41}$ peptides.

Supplementary Fig. 4. Analysis of P14 $CD8^+$ T cell functionality and the induced $Foxp3^+$ Treg cell population primed with ARM-infected DCs.

[a] The proliferation and division of P14 T cells cocultured with UN-BMDCs or ARM-infected BMDCs were analyzed by CellTrace dye staining. The bar graph presents the percentage of the cell populations in each division number.

[**b**] The potential cytokine secretion of P14 T cells was analyzed by intracellular cytokine staining and CellTrace dye staining. The kinetics of effector cytokine expression during P14 T cell proliferation were analyzed (top two FACS plots and bar graph). The expression levels of IFN-γ, IL-2, TNF-α and granzyme B in activated $CD44^{hi}$ proliferating P14 T cells were investigated (bottom two FACS plots and bar graph).

[**c**] The total amount of each cytokine expressed in the supernatant of P14-BMDC cocultures was measured by a multiplex bead assay.

[**d**] Conversion ratio of $Foxp3^+$ Treg cells in a coculture containing $CD4^+$ T cells with UN-BMDCs or ARM-infected BMDCs in each condition.

[**e**] The proliferation and division of P14 T cells cocultured with *ex vivo* SPDCs from uninfected or ARM-infected mice were analyzed. The bar graph presents the percentages of the cell populations in each division number.

[**f**] The cytokine-secreting potential of P14 T cells was analyzed. The kinetics of effector cytokine expression during P14 T cell proliferation were analyzed (top two FACS plots and bar graph). The expression levels of IFN-γ, IL-2, TNF-α and GzmB in activated $CD44^{hi}$ proliferating P14 T cells were investigated (bottom two FACS plots and bar graph).

[**g**] The total amount of each cytokine expressed in the supernatant of P14-SPDC cocultures was measured by a multiplex bead assay.

[**h**] Conversion ratio of $Foxp3^+$ Treg cells derived from $CD4^+$ T cells cocultured with SPDCs from uninfected or ARM-infected mice in each condition.

The bar graphs show the means ± SDs (6 mice in each group). Each experiment was repeated

twice. The *p* values in the figures indicate the following: $^*P < 0.05$; $^{**}P < 0.01$; $^{**}P < 0.01$; $^{***}P < 0.001$.

Supplementary Fig. 5. Analysis of P14 CD8$^+$ T cell functionality after priming with CD11b$^+$ SPDCs selectively expanded by GM-CSF-expressing tumor cells during chronic CL13 infection.

[a] Experimental scheme for manipulating CD11b$^+$ DCs in the spleen with GM-CSF expressing B16F10 cells.

[**b]** Expansion of each subset of SPDCs with B16F10 cells engineered to express GM-CSF or Flt3L. The percentages (top, flow cytometry plot) and numbers (bottom, bar plot) of cDC1s, cDC2s and pDCs in the spleen on Day 9 after injection of each tumor cell line. The injection of B16F10-GM-CSF cells selectively expanded the CD11b$^+$ cDC2 population.

[**c]** Proliferation and effector cytokine expression analysis of P14 cells primed with CD11b$^+$ SPDCs expanded by injection with GM-CSF-expressing B16F10 cells.

[**d]** Analysis of induced Foxp3$^+$ Treg cells derived from CD4$^+$ T cells cocultured with CD11b$^+$ SPDCs expanded by injection of B16F10-GMCSF cells.

The bar graphs show the means ± SDs (3 mice in each group). Each experiment was repeated twice. The *p* values in the figures indicate the following: $^*P < 0.05$; $^{**}P < 0.01$; $^{**}P < 0.01$; $^{***}P < 0.001$.

Supplementary Fig. 6. Functional analysis of P14 T cells primed with $CD11b^+$ myeloid DCs in the context of PD-L1 blockade.

[a] The blockade of PD-L1 on the surface of BMDCs after treatment with a purified anti-PD-L1 antibody was verified by flow cytometry analysis.

[**b]** The kinetics of the expression of various molecules, such as PD-1, IFN-γ, IL-2 and TNF-α, in P14 T cells cocultured with BMDCs were investigated.

[c] Upon coculture with BMDCs, the frequency of each effector cytokine-expressing $CD44^{hi}$ activated P14 T cell population was analyzed in each condition.

[**d]** The total amount of each effector cytokine in the supernatant of P14-BMDC cocultures was estimated by a multiplex bead assay.

[**e]** The blockade of PD-L1 on the surface of SPDCs after treatment with purified anti-PD-L1 antibody was verified by flow cytometry analysis.

[**f]** The kinetics of the expression of various molecules, such as PD-1, IFN-γ, IL-2 and TNF-α, in P14 T cells cocultured with SPDCs were assessed.

[**g]** Upon coculture with SPDCs, the frequency of each effector cytokine-expressing $CD44^{hi}$ activated P14 T cell population was analyzed in each condition.

[**h]** The total amount of each effector cytokine in the supernatant of P14-SPDC cocultures was estimated by a multiplex bead assay.

The bar graphs show the means ± SDs (3 mice in each group). Each experiment was repeated twice. The *p* values in the figures indicate the following: *P < 0.05; **P <0.01; **P < 0.01;

***P < 0.001.

Supplementary Fig. 7. Functional analysis of OT-I CD8$^+$ T cells primed with ARM-infected DCs.

[a] The *in vitro* proliferation and division of OT-I T cells cocultured with OVA-pulsed naïve or ARM-infected SPDCs were analyzed by CellTrace dye staining. The bar graph presents the percentages of the cell populations in each division number.

[**b]** The potential cytokine secretion of OT-I T cells cocultured with OVA-pulsed naïve or ARM-infected SPDCs was analyzed by intracellular cytokine staining and CellTrace dye staining. The kinetics of effector cytokine expression during OT-I T cell proliferation were analyzed (top two FACS plots and bar graph). The expression levels of IFN-γ, IL-2, and TNF-α in activated CD44^{hi} proliferating OT-I T cells were investigated (bottom two FACS plots and bar graph).

[**c]** Analysis of the proliferation and division of adoptively transferred OT-I T cells in the spleen of uninfected or ARM-infected mice after VV-OVA coinfection. The bar graph presents the percentages of the cell populations in each division number.

[**d]** The cytokine-secreting potential of adoptively transferred OT-I T cells was analyzed by intracellular cytokine staining and CellTrace dye staining. The kinetics of effector cytokine expression during OT-I T cell proliferation were analyzed (top three FACS plots and bar graphs). The expression levels of IFN-γ, IL-2, and TNF-α in activated CD44^{hi} proliferating OT-I T cells were investigated (bottom three FACS plots and bar graphs).

The bar graphs show the means ± SDs (6 mice in each group). Each experiment was repeated twice. The *p* values in the figures indicate the following: $*P < 0.05$; $**P < 0.01$; $**P < 0.01$; $***P < 0.001$
